# Changes in prevalence and sociodemographic correlates of tobacco and nicotine use in Finland during the COVID-19 pandemic

**DOI:** 10.1093/eurpub/ckad104

**Published:** 2023-07-03

**Authors:** Sebastián Peña, Katja Ilmarinen, Laura Kestilä, Otto Ruokolainen, Hanna Ollila, Suvi Parikka, Sakari Karvonen

**Affiliations:** Department of Public Health and Welfare, Finnish Institute for Health and Welfare, Helsinki, Finland; Department of Public Health and Welfare, Finnish Institute for Health and Welfare, Helsinki, Finland; Department of Public Health and Welfare, Finnish Institute for Health and Welfare, Helsinki, Finland; Department of Public Health and Welfare, Finnish Institute for Health and Welfare, Helsinki, Finland; Department of Public Health and Welfare, Finnish Institute for Health and Welfare, Helsinki, Finland; Department of Public Health and Welfare, Finnish Institute for Health and Welfare, Helsinki, Finland; Department of Public Health and Welfare, Finnish Institute for Health and Welfare, Helsinki, Finland

## Abstract

**Background:**

The impact of the coronavirus disease 2019 (COVID-19) pandemic on tobacco and nicotine use remains debated. We examined whether the prevalence of tobacco and nicotine use and nicotine-replacement therapy (NRT) changed during the COVID-19 pandemic and whether changes differed by sociodemographic groups.

**Methods:**

Repeated cross-sectional study of three national surveys in Finland (2018, 2019 and 2020; *n* = 58 526 adults aged 20 and over). Outcomes were daily and occasional smoking, smokeless tobacco (snus) use, e-cigarettes use, total tobacco or nicotine use and NRT use. We examined changes for each outcome by sex, age, educational tertiles, marital status, mother tongue and social participation.

**Results:**

Daily smoking decreased among males by 1.15 percentage points (pp) [95% confidence interval (CI) −2.10 to −0.20] between 2018 and 2020 and 0.86 pp among females (95% CI −1.58 to −0.15). Daily snus use remained stable in both sexes. Daily e-cigarette use was below 1% and remained stable. We found weak evidence of a reduction in total tobacco or nicotine use between 2018 and 2020 (males −1.18 pp, 95% CI −2.68 to 0.32 and females −0.8 pp, 95% CI −1.81 to 0.22). NRT use remained stable. Snus and NRT use decreased among 60- to 74-year-olds but remained stable in other age groups. We did not find evidence of interactions by subgroup for other outcomes.

**Conclusions:**

Daily smoking decreased in Finland between 2018 and 2020, but other forms of tobacco use did not experience a reduction. The COVID-19 pandemic does not seem to have altered the sustained reduction of smoking in Finland, although substantial sociodemographic differences persist.

## Introduction

The coronavirus disease 2019 (COVID-19) pandemic continues to impact population health and well-being worldwide. By 21 June 2023, there were more than 768 million cases of COVID-19 and 6.9 million deaths reported worldwide.^1^ Tobacco use is a leading risk factor for death and disability worldwide^2^ as well as a risk factor for COVID-19 disease severity.^3^

Changes in tobacco and nicotine use during the COVID-19 pandemic, which might have profound long-term health consequences, could emerge from contrasting forces. On one hand, several factors pressured reductions in tobacco and nicotine use. First, social distancing policies could reduce social opportunities for tobacco use by reshaping social interactions and disrupting routines.^4^ Second, travelling restrictions could restrict tobacco availability due to diminished passenger imports and illicit supply of tobacco and nicotine products [e.g. reducing imports of smokeless tobacco (snus) from Sweden to Finland]. Third, increasing unemployment rates and job insecurity^5^ could reduce disposable income and affordability of tobacco and nicotine products. Fourth, the pandemic might have increased health awareness and prompted individuals to reduce or quit smoking, switch to other forms of tobacco or nicotine use or combine it with medicinal products.^6^^,^^7^

On the other hand, pressures to increase tobacco and nicotine use include the experience of emotional distress, social isolation and boredom.^6^^,^^8^ In addition, a potential protective effect of smoking on the risk of COVID-19 has received significant media attention, which together with intensified marketing of e-cigarettes in some settings could potentially erode decades of public health awareness on tobacco-related harms.^9^ As lower socioeconomic groups report higher prevalences of tobacco use^10^^,^^11^ and experience greater tobacco-related harm,^12^ it is crucial to explore whether changes in tobacco and nicotine use differ by sociodemographic characteristics.

Most studies examining changes in the use of products containing tobacco and/or nicotine (such as cigarettes, nicotine pouches or e-cigarettes) and medicinal nicotine products during the COVID-19 pandemic have been based on volunteer samples (primarily social media users) or have assessed smoking before the pandemic retrospectively,^13–16^ being at high risk of selection bias (due to voluntary participation) and recall bias.

To our knowledge, two studies in the UK and one in South Asian countries have reported estimates with data collected pre and post-pandemic among adults. In the UK, current smoking decreased during the COVID-19 pandemic from 15.1% in 2017–19 to 12.1% in April 2020.^17^ Another UK study did not find evidence of changes in smoking prevalence.^7^ In the same sample, the authors found weak evidence of increasing use of nicotine-replacement therapy (NRT) products during the COVID-19 pandemic.^18^ In South Asian countries, current smoking decreased from 24.4% to 14.5%.^19^ Overall, little information from nationally representative samples exists on how the COVID-19 pandemic has affected tobacco and nicotine use in different population groups. Moreover, these previous studies have primarily focused on smoking, without providing a comprehensive picture of tobacco and nicotine use and NRT medicinal products in the population.

Finland approached the COVID-19 pandemic with two different strategies. Between March and May 2020, the Finnish Government introduced a nationwide closure of schools and public facilities, prohibited public gatherings of more than 10 people, introduced remote work for all public sector employees, and instituted the closure of restaurants and bars (except for take away and personnel canteens). In May 2020, the country transitioned to a so-called ‘hybrid strategy’ which eased previous restrictions and instead introduced targeted regional measures and broad testing-tracing-isolation actions.^20^ Supermarkets and grocery stores, where most tobacco products are sold, remained open during the pandemic.

In this study, we examined whether (i) the prevalence of smoking, use of snus, e-cigarettes with and without nicotine, total tobacco and nicotine use and use of NRT products changed during the COVID-19 pandemic and (ii) these prevalence changes varied by sociodemographic factors.

## Methods

### Study design

The study has a repeated cross-sectional design. Study populations were permanent residents in Finland aged 20 and over from the FinSote surveys (2018, 2019 and 2020). We reported the results in accordance with the Strengthening the Reporting of Observational Studies in Epidemiology (STROBE) statement.^21^ The study was registered in the OSF framework (10.17605/osf.io/vpsyz). We made minor changes to the pre-registered analysis, which are described in the Supplementary Appendix.

### Data sources

We used data from three cross-sectional population health surveys in Finland. FinSote 2018 was a national survey of the Finnish population aged 20 years and over. The sampling frame was the Population Register of Statistics Finland.^22^ The survey was based on stratified random sampling, one stratum for each region in mainland Finland (2300 adults aged 20–74 and 1000 adults aged 75+, total sample size 59 400). Data were collected between October 2017 and March 2018. Participants received a self-administered questionnaire in Finnish, Swedish, English and Russian, which could be returned on paper or filled in electronically. The analytical sample was 26 422 participants (participation rate 45%).^22^

FinSote 2019 was a national survey of the Finnish population aged 15 and over, implemented in conjunction with the European Health Information Survey round 3. A random sample of 15 000 individuals was drawn from the Population Register of Statistics Finland. Data were collected between September 2019 and January 2020. Participants received a self-administered questionnaire available in Finnish, Swedish and English, which could be returned on paper or filled in electronically. Participation rate was 44%, which after restricting to participants aged 20 and over, resulted in an analytical sample of 5943 participants.^23^

FinSote 2020 was a national survey of the Finnish population aged 20 and over. The sampling frame was the Digital and Population Data Services Agency, created in January 2020 after the merger between the Population Register of Statistics Finland and local register offices.^24^ The survey was based on a stratified random sample of the 22 well-being areas of mainland Finland (2000 adults aged 20–74, 800 adults aged 75+, total sample size 61 600). Data were collected from September 2020 to February 2021. Participants received a self-administered questionnaire in Finnish, Swedish, English and Russian, which could be returned on paper or filled in electronically. The analytical sample was 28 199 participants (participation rate 46%).

### Outcomes

We examined five primary outcomes: (i) prevalence of daily and occasional smoking, (ii) daily and occasional snus use, (iii) use of e-cigarettes with nicotine, (iv) use of e-cigarettes without nicotine and (v) total use of tobacco or nicotine products. As a secondary outcome, we reported the prevalence of NRT product use. The Supplementary Appendix includes a detailed description of the exact questions and definitions. Questions on other outcomes than smoking were not asked for those aged 75+; we, therefore, report smoking for people aged 20 and older and other outcomes for participants aged 20–74 years old.

#### Smoking status

Smoking status in 2018 and 2020 was assessed by asking participants about their current smoking. We classified smoking as never smokers, former smokers, current occasional smokers and current daily smokers. In 2019, questions were slightly different. The first question asked about their present smoking, followed by a question on whether they have ever smoked daily for a period of at least 1 year. We used the first question to categorize daily and occasional smokers and the second to classify participants into former and never smokers.

#### Snus use

Snus use was categorized as never users, former users, current occasional users and current daily users.

#### E-cigarettes with and without nicotine

We compared data from 2018 and 2020, as FinSote 2019 did not have separate questions on e-cigarettes with and without nicotine. E-cigarettes use (with and without nicotine separately) was categorized as never users, former users, current occasional users and current daily users.

#### Total tobacco and nicotine products use

We reported the current daily use of any tobacco or nicotine product (0 = no use, 1 = reported either smoking, using snus or e-cigarettes with nicotine).

#### NRT products

We used data from 2018 and 2020 because FinSote 2019 survey did not have questions on NRT products. NRT use was classified as never users, former users, current occasional users and current daily users.

### Sociodemographic characteristics

We included several sociodemographic characteristics to provide a complete picture of how tobacco and nicotine use varies in the Finnish population. We included age [categorized as 20–39, 40–59 and 60+ for smoking, 60–74 for other outcomes, sex (male, female), educational level (years of education divided into tertiles), marital status (married, in a registered partnership or cohabiting versus single, separated or widowed), mother tongue (Finnish, Swedish and others) and participation in social activities (no participation, occasional participation and active participation). Data on age, sex and mother tongue were obtained from national registries.

### Statistical analysis

We calculated prevalence estimates with 95% confidence intervals (CIs) for each outcome using ordered logistic regression (given the ordered nature of the outcomes). We reported prevalences stratified by sex. For this purpose, we ran a model for each outcome (six models) adjusted for age, sex and survey wave and used predictive margins with an interaction term for survey wave and sex to obtain stratified age-adjusted prevalences for each survey wave. The variance was estimated using the linearization method. In addition, we carried out pairwise comparisons of margins to estimate the prevalence difference between 2018 and 2020 and their 95% CI. To account for differences in age and sex distribution, we used 2020 as the reference population. Results should thus be interpreted as ‘the prevalence in a given year if age and sex distributions were the same as in 2020’. We tested a linear calendar time trend to examine whether the outcome changed linearly over time.

For smoking, differences in survey questionnaires between 2018–20 and 2019 resulted in an inconsistent prevalence of never and former smokers across years, which violated the proportional odds assumption (see the Supplementary Appendix for details). To account for this, we combined never and former smokers for the smoking outcome.

We reported age and sex-adjusted predictive margins for each subgroup. We combined both sexes because we did not find interactions between sex and survey year. We also tested for change over time by using the adjusted Wald test in an age- and sex-adjusted model with interaction terms between survey year (as a continuous variable) and age, sex, marital status, educational level, mother tongue and participation in social activities, introduced one at a time (i.e. six models for each outcome). We observed statistically significant interactions between age and survey year for snus use and NRT products. Therefore, the main model included additionally an interaction term between age and survey year.

We excluded participants with missing data in each model. As there were no missing data on sex and age, prevalence estimates exclude participants with missing data for that specific outcome (overall prevalence) or specific outcome and sociodemographic variable. We used inverse probability weights to account for non-participation (see Supplementary Appendix for further details). All analyses took the complex survey design into account. In post hoc sensitivity analyses, we report smoking prevalence for those aged 20–74. We used R version 4.1.1 and Stata SE 17 for the analyses. An annotated statistical code is available in the Supplementary Appendix.

## Results

We analyzed 58 526 participants aged 20 and over with data on smoking (38 675, aged 20–74 years). The proportion of missing data ranged from 0% to 8.2% and was similar between study years (Supplementary table S1). Of the analytical sample, 51.4% were females; 35.3% were separated, single or widowed; had 13.7 years of education on average; 93.5% spoke Finnish as their mother tongue; and 50.6% reported no participation in social activities. These characteristics remained stable between 2018 and 2020, except for an increase in reported lack of participation in social activities in 2020 (table 1).

**Table 1:** Characteristics of 58 526 participants of FinSote 2018–20

	2018	2019	2020
*n*	25 451	5675	27 400
Sex, % female	51.9	51.0	51.7
Mean age (SD)	51.8 (18.5)	51.6 (18.4)	52.0 (18.7)
Marital status, % separated, single or widowed	34.8	34.4	36.0
Mean years of education (SD)	13.7 (4.1)	14.3 (5.1)	13.7 (3.9)
Mother tongue, %			
Finnish	93.6	94.5	93.3
Swedish	4.6	5.1	4.9
Other	1.8	0.4	1.9
Participation in social activities, %			
Active	27.9	27.8	24.7
Occasional	22.7	25.8	23.0
No participation	49.3	46.5	52.3

Notes. Data are percentages or mean (SD). All values take the complex sampling design into account.

### Prevalence of tobacco and nicotine use

Table 2 shows age-adjusted prevalences for all outcomes between 2018 and 2020 stratified by sex. Smoking was the most prevalent form of tobacco and nicotine use in Finland, followed by snus use in both sexes. We found a declining linear trend for smoking status (*P* = 0.020), but not for the other outcomes (Supplementary table S2). Among males, the prevalence of daily smoking experienced a reduction from 13.7% in 2018 to 12.5% in 2020, while occasional smoking experienced a smaller reduction from 8.1% in 2018 to 7.6% in 2020. Females also experienced a reduction in daily smoking (9.9% in 2018 vs. 9.0% in 2020) and occasional smoking (6.3% in 2018 vs. 5.9% in 2020). This translates into an overall decline in daily smoking of −1.0 percentage points (pp) (95% CI −1.83 to −0.17) from 2018 to 2020 and a decline in occasional smoking of −0.48 pp (95% CI −0.88 to −0.09) (table 3). Prevalence of daily and occasional snus use remained at similar levels between 2018 and 2020 for both males and females. The daily use of e-cigarettes with and without nicotine was overall very infrequent (<1%) over the study years and showed a stable prevalence. Total tobacco or nicotine use decreased among males from 19.2% to 18.0% between 2018 and 2020 (prevalence difference −1.18 pp, 95% CI −2.68 to 0.32) and among females from 11.9% to 11.1% between 2018 and 2020 (prevalence difference −0.8 pp, 95% CI −1.81 to 0.22). The use of NRT products remained stable over the study period.

**Table 2:** Model-adjusted prevalences and 95% CIs of tobacco and nicotine use in Finland by sex, 2018–20

	Males			Females		
	2018	2019	2020	2018	2019	2020
Smoking (*n* = 58 526)						
Daily	13.7 (12.7–14.6)	13.0 (12.0–14.1)	12.5 (11.8–13.2)	9.9 (9.1–10.6)	9.4 (8.6–10.2)	9.0 (8.4–9.6)
Occasional	8.1 (7.5–8.7)	7.8 (7.2–8.4)	7.6 (7.1–8.1)	6.3 (5.9–6.8)	6.1 (5.6–6.6)	5.9 (5.5–6.3)
Never and former smoker	78.2 (76.9–79.5)	79.1 (77.7–80.5)	79.9 (78.9–80.8)	83.8 (82.8–84.8)	84.5 (83.4–85.7)	85.1 (84.3–85.9)
Snus use (*n* = 38 754)						
Daily user	3.7 (3.3–4.2)	3.0 (2.6–3.4)	3.6 (3.2–4.0)	1.5 (1.3–1.7)	1.1 (1.0–1.3)	1.5 (1.3–1.7)
Occasional user	3.9 (3.4–4.4)	3.1 (2.7–3.5)	3.8 (3.3–4.2)	1.5 (1.3–1.7)	1.1 (0.9–1.2)	1.5 (1.3–1.7)
Former user	21.3 (19.9–22.8)	15.7 (14.5–17.0)	21.0 (20.0–22.0)	6.6 (5.9–7.2)	4.6 (4.1–5.2)	6.5 (6.0–7.1)
Never user	71.0 (69.4–72.7)	78.2 (76.6–79.8)	71.7 (70.5–72.9)	90.4 (89.6–91.3)	93.2 (92.4–93.9)	90.5 (89.8–91.2)
E-cigarettes with nicotine (*n* = 33 710)						
Daily user	0.9 (0.7–1.1)		0.9 (0.7–1.1)	0.5 (0.4–0.6)		0.5 (0.4–0.6)
Occasional user	1.3 (1.1–1.6)		1.3 (1.1–1.6)	0.7 (0.6–0.9)		0.7 (0.6–0.9)
Former user	20.0 (18.5–21.4)		19.7 (18.6–20.8)	9.4 (8.6–10.3)		9.3 (8.6–10.0)
Never user	77.8 (76.2–79.3)		78.0 (76.9–79.2)	89.3 (88.4–90.2)		89.5 (88.7–90.2)
E-cigarettes without nicotine (*n* = 33 562)						
Daily user	0.1 (0.1–0.2)		0.1 (0.1–0.2)	0.1 (0.0–0.1)		0.1 (0.0–0.1)
Occasional user	0.6 (0.4–0.8)		0.6 (0.4–0.8)	0.3 (0.2–0.4)		0.3 (0.2–0.4)
Former user	18.5 (17.0–19.9)		18.2 (17.1–19.3)	8.8 (8.0–9.7)		8.7 (8.0–9.4)
Never user	80.8 (79.3–82.3)		81.1 (80.0–82.2)	90.8 (89.9–91.6)		90.9 (90.2–91.6)
Any daily use of tobacco or nicotine products (*n* = 34 212)						
Any daily use	19.2 (17.7–20.6)		18.0 (16.9–19.1)	11.9 (10.9–12.9)		11.1 (10.3–11.9)
No daily use	80.8 (79.4–82.3)		82.0 (80.9–83.1)	88.1 (87.1–89.1)		88.9 (88.1–89.7)
Nicotine-replacement therapy products (*n* = 33 680)						
Daily user	1.9 (1.6–2.1)		1.9 (1.6–2.1)	1.2 (1.1–1.4)		1.2 (1.1–1.4)
Occasional user	4.1 (0.4–0.8)		4.1 (0.4–0.8)	2.6 (2.3–2.9)		2.6 (2.3–2.9)
Former user	20.8 (17.0–19.9)		21.2 (17.1–19.3)	11.5 (10.7–12.4)		11.8 (11.1–12.5)
Never user	73.3 (71.6–74.9)		72.9 (71.7–74.1)	84.7 (83.6–85.7)		84.3 (83.4–85.2)

Notes. Data are percentages (95% CI). Prevalence estimates are age-adjusted and obtained using 2020 as the reference population. All prevalences incorporate the complex sampling design and represent the Finnish population. Data on smoking were available for the whole sample (aged 20 and over). Data on snus, e-cigarettes with or without nicotine and NRT products were available for those aged 20–74 years old.

**Table 3:** Prevalence difference for daily and occasional use of tobacco and nicotine products between 2018 and 2020

	Overall		Males		Females	
	Prevalence difference	95% CI	Prevalence difference	95% CI	Prevalence difference	95% CI
Smoking (*n* = 58 526)						
Daily	−1.002	−1.83 to −0.17	−1.147	−2.1 to −0.2	−0.865	−1.58 to −0.15
Occasional	−0.48	−0.88 to −0.09	−0.512	−0.93 to −0.09	−0.455	−0.83 to −0.08
Never and former smoker	1.48	0.26 to 2.71	1.659	0.29 to 3.03	1.32	0.23 to 2.41
Snus use (*n* = 38 754)						
Daily user	−0.08	−0.2 to 0.05	−0.141	−0.28 to 0.00	−0.023	−0.14 to 0.09
Occasional user	−0.07	−0.22 to 0.07	−0.134	−0.31 to 0.04	−0.019	−0.14 to 0.1
Former user	−0.18	−1.23 to 0.87	−0.374	−1.89 to 1.14	−0.038	−0.62 to 0.54
Never user	0.34	−0.98 to 1.65	0.649	−1.16 to 2.46	0.081	−0.73 to 0.89
E-cigarettes with nicotine (*n* = 33 710)						
Daily user	−0.01	−0.05 to 0.04	−0.008	−0.06 to 0.04	−0.006	−0.04 to 0.03
Occasional user	−0.01	−0.08 to 0.06	−0.012	−0.09 to 0.06	−0.009	−0.07 to 0.05
Former user	−0.18	−1.38 to 1.01	−0.24	−1.79 to 1.31	−0.129	−0.97 to 0.71
Never user	0.2	−1.1 to 1.5	0.259	−1.42 to 1.94	0.144	−0.79 to 1.08
E-cigarettes without nicotine (*n* = 33 562)						
Daily user	−0.001	−0.01 to 0.01	−0.001	−0.01 to 0.01	−0.001	−0.01 to 0.00
Occasional user	−0.01	−0.04 to 0.03	−0.007	−0.05 to 0.03	−0.005	−0.03 to 0.02
Former user	−0.21	−1.42 to 0.99	−0.28	−1.85 to 1.29	−0.151	−0.99 to 0.69
Never user	0.22	−1.02 to 1.47	0.289	−1.33 to 1.91	0.157	−0.72 to 1.03
Any daily use of tobacco or nicotine products (*n* = 34 212)						
Any daily use	−0.99	−2.24 to 0.27	−1.18	−2.68 to 0.32	−0.8	−1.81 to 0.22
No daily use	0.99	−0.27 to 2.24	1.18	−0.32 to 2.68	0.8	−0.22 to 1.81
Nicotine-replacement therapy products (*n* = 33 680)						
Daily user	0.007	−0.07 to 0.08	0.0	−0.07 to 0.07	0.014	−0.06 to 0.09
Occasional user	0.02	−0.15 to 0.2	0.012	−0.17 to 0.19	0.035	−0.13 to 0.2
Former user	0.32	−0.84 to 1.48	0.356	−1.07 to 1.78	0.264	−0.63 to 1.16
Never user	−0.35	−1.76 to 1.05	−0.368	−2.05 to 1.31	−0.314	−1.45 to 0.82

### Sociodemographic differences in tobacco and nicotine use

**Figure 1:**
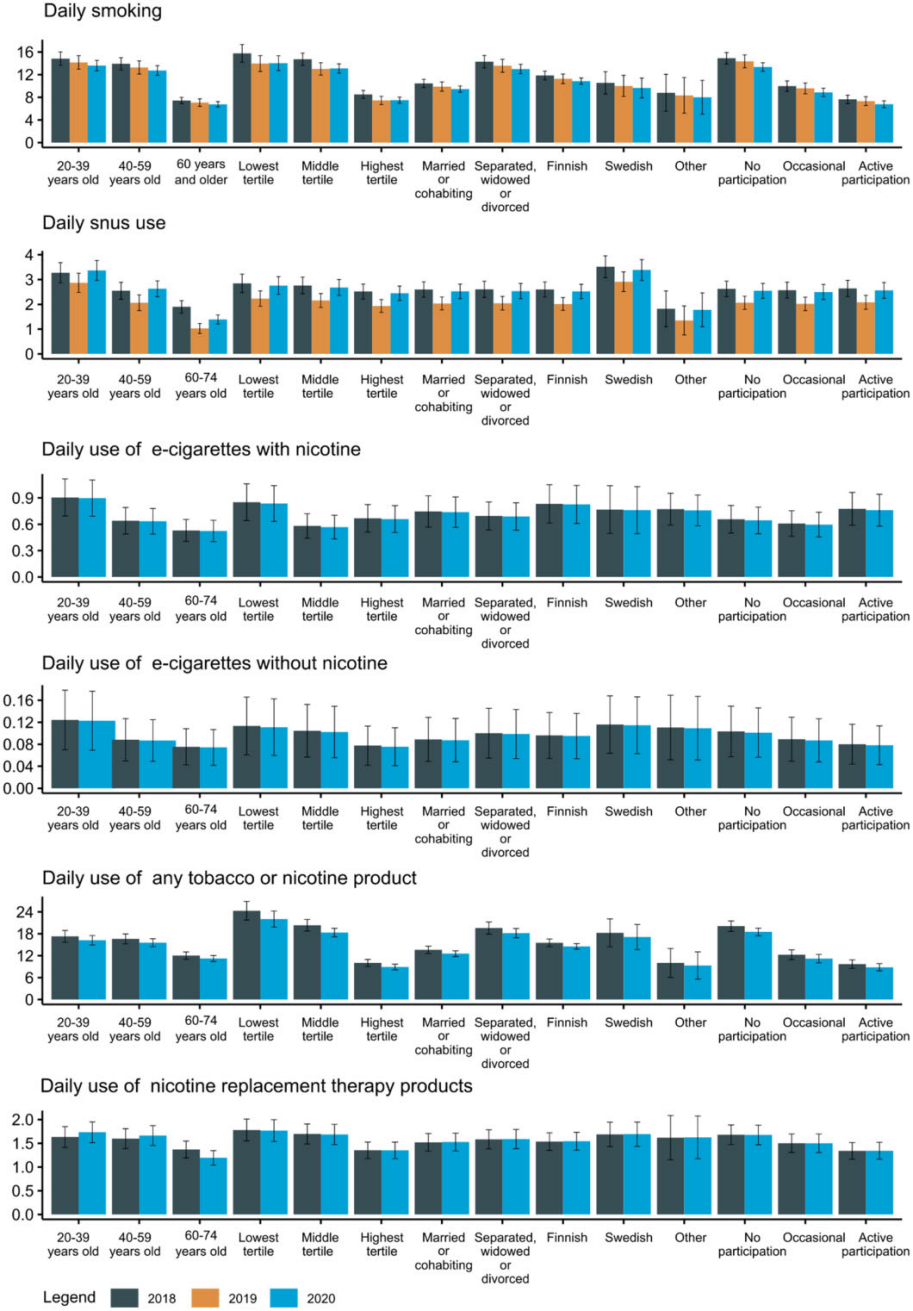
Model-adjusted prevalences of daily tobacco and nicotine use in Finland by age groups, educational tertiles, marital status, mother tongue and social participation, 2018–20. Note: Estimates are predicted means from ordered logistic regression models. Data on smoking were available for those aged 20 years and older. Data on snus, e-cigarettes with or without nicotine and NRT products were available for those 20–74 years old

We observed clear sociodemographic differences in daily smoking. Males, people aged 20–39 years old, in the lowest educational tertile, separated, divorced or widowed, Finnish speaking and reporting no participation in social activities reported the highest prevalence of daily smoking in their respective categories in all years (figure 1 and Supplementary tables S4–S9). Daily snus use was higher among males, those aged 20–39 years old and who spoke Swedish as their mother tongue. We observed small sociodemographic differences in the use of e-cigarettes. Sociodemographic differences in total tobacco or nicotine use were similar to those in the use of smoking. There were very small sociodemographic differences in the use of NRT products.

We only observed interactions between age and survey year for snus use and NRT use, revealing a decrease in both daily and occasional use of snus and NRT products among 60- to 74-year-olds, while it remained stable in the other age groups (Supplementary tables S5 and S8). We did not find evidence of interactions by subgroup for the other outcomes (Supplementary table S2). Sociodemographic differences for occasional use were very similar to daily use for all outcomes (Supplementary figure S1).

In post hoc sensitivity analyses, we observed similar results for smoking among those aged 20–74 years old (Supplementary table S3).

## Discussion

We examined the prevalence of tobacco and nicotine use in Finland between 2018 and 2020. We observed a steady reduction in daily and occasional smoking in both sexes. We did not observe changes in daily or occasional snus use and e-cigarette use. We found weak evidence of a reduction in total tobacco and nicotine use. NRT product use remained stable over the study period. We found substantial sociodemographic differences in smoking, snus use and total tobacco or nicotine use. Snus and NRT products use decreased among 60–74 years old and remained stable in other age groups. Other socioeconomic differences remained stable over the study period.

We found a reduction in daily and occasional smoking between 2018 and 2020 in both sexes. Our results are consistent with studies reporting reductions in smoking prevalence during the COVID-19 pandemic in a recent systematic review,^13^ in the UK,^17^ in four South Asian countries,^19^ as well as a 33% reduction in tobacco compounds in wastewater in Greece.^25^ One potential explanation for the reduction observed in Finland is secular trends. Smoking prevalence has consistently declined in Finland, primarily among males. Daily smoking decreased among males from 37% to 17% between 1978 and 2016.^10^ The observed reduction in smoking prevalence among females in our study is in contrast with a relatively stable prevalence of daily smoking of ∼15% between 1978 and 2016.^10^ Finland has a longstanding and robust tobacco control policy, which might have been able to buffer some of the effects of the COVID-19 pandemic. In addition, a menthol ban came into effect in the European Union in May 2020, which could have supported smoking reduction and cessation efforts.^26^ As discussed above, reductions in tobacco affordability and disruptions in social routines may have also contributed to the observed reduction.

We did not find evidence of changes in daily and occasional snus use from 2018 to 2020. This is somewhat surprising, as travelling between Finland and Sweden was restricted during the pandemic. However, the strictest restrictions happened during the first wave (March to June 2020) and were partially eased by the time of FinSote 2020 data collection. Limits for traveller imports of snus are also large (allowance is 1 kg per calendar day, lasting generally 4 months of use), allowing sustaining snus use without travelling frequently to Sweden, in addition to illegal purchases which likely persisted during the pandemic. Comparison with previous studies is limited as, to our knowledge, the only previous study described quitting behaviours during the COVID-19 lockdown among smokeless tobacco users in India. The study reported that 32.1% of smokeless tobacco users have quit use during the lockdown.^27^

We did not find evidence of prevalence changes in e-cigarettes with or without nicotine. This is partly explained by the very low prevalence of e-cigarette use, which is subject to strict regulations.^28^^,^^29^ Studies in the UK, USA and Canada have reported reductions in e-cigarettes in the general population,^14^^,^^17^^,^^30^ young adults^31^ and adolescents,^32^ while a recent Italian study showed an increase in prevalence from 8.1% to 9.1% among adults.^33^

We did not find evidence of changes in NRT products use. This is in contrast with a UK study which found weak evidence of higher odds of NRT use during the COVID-19 pandemic.^18^ In the USA, 26% of smokers and 41% of e-cigarette users attempted to quit because of COVID-19.^34^ Taking together a reduction in smoking prevalence, these findings might indicate increased attempts to quit or use NRT products instead of tobacco.

We found sustained sociodemographic differences by sex, age, educational levels, marital status, mother tongue and participation in social activities. This is consistent with studies in Finland, Europe and the USA.^10^^,^^11^^,^^35^^,^^36^ Regarding change over time, we observed interactions between age and survey year for the use of snus and NRT products, but not for smoking or e-cigarette use. While recent studies in Finland show that smokers have ‘softened’ (i.e. are more responsive to tobacco control interventions), lower socioeconomic groups experience lower quitting rates and consume a higher daily number of cigarettes.^37^ This could be a result of the higher availability of tobacco products in lower socioeconomic areas, as a recent study suggest,^38^ or due to other social and environmental factors that reduce the likelihood that smokers in lower socioeconomic groups benefit from tobacco control interventions.^7^^,^^17^

Strengths of our study include (i) the use of national population surveys from Finland, (ii) a sampling frame that includes people living in institutions and conscripts. The sampling frame is also consistent in the study years (the change in 2020 was merely administrative), (iii) the inclusion of a wide variety of forms of tobacco and nicotine use and (iv) the use of a comparable questionnaire and data collected prior to the COVID-19 pandemic, which reduces the risk of recall bias.

However, some limitations are noted. We explored a relatively narrow time frame of three consecutive years. This means we cannot robustly disentangle the effects of the COVID-19 pandemic from secular trends in tobacco and nicotine use. Another important limitation is the lack of data on smoking intensity. A recent meta-analysis showed that 21% of smokers increase their smoking intensity,^13^ which might offset population benefits of reductions in smoking. We also lacked data on heated tobacco products, an emerging tobacco product with a similar prevalence to e-cigarettes in Finland.^39^ This means that our estimates on total tobacco and nicotine use are likely underestimated. Finally, non-participation in FinSote surveys was relatively high and posed a threat to internal validity. We used inverse probability weights to account for non-participation and reduce the risk of selection bias, but we cannot rule out that some selection bias persists.

Our study has important public health implications. First, our results reinforce the importance of a robust tobacco control policy, which might also make countries more resilient to societal shocks such as the COVID-19 pandemic. However, it should be noted that despite the observed reduction, Finland failed to meet its interim target of 10% of daily use of tobacco and nicotine products by 2020^40^ in both men and women and, at this rate, will not reach the goal of 5% of daily use of tobacco and nicotine products by 2030. Second, substantial sociodemographic differences persist, suggesting the need for targeted interventions in higher prevalence groups as well as structural policies addressing the social, commercial, political and environmental determinants of health.

In conclusion, daily and occasional smoking decreased in Finland between 2018 and 2020. Other forms of tobacco use did not experience a similar reduction. We found large sociodemographic differences, which remained stable over the study period. Future research could explore longer-time trends, triangulate data from sales and other sources to disentangle the effects of the COVID-19 pandemic from secular trends in tobacco use or examine changes in dual use of tobacco and nicotine products.

## Supplementary Material

ckad104_Supplementary_Data

## Data Availability

The data used in this study are not publicly available but can be accessed with a research proposal and an approved user authorization application from the Finnish Social and Health Data Permit Authority (Findata). For more information, please visit https://findata.fi/en/ The coronavirus disease 2019 (COVID-19) pandemic could impact tobacco and nicotine use, with long-term health and well-being consequences. Most studies are based on cross-sectional assessments of convenience samples (such as social media users), which are subject to a high risk of selection and information bias. Little information exists from repeated nationally representative studies. We examined whether the prevalence of several forms of tobacco and nicotine use and medicinal products changed during the COVID-19 pandemic and whether these changes vary by sociodemographic groups. We found a reduction in daily and occasional smoking, yet other forms of tobacco use remained stable. Sociodemographic differences persisted over the time period. Our findings suggest that a robust tobacco control policy, such as the one implemented by Finland over decades, might have been able to buffer some of the effects of the COVID-19 pandemic on tobacco use.
