# Supplementary material for: Changes in prevalence and sociodemographic correlates of tobacco and nicotine use in Finland during the COVID-19 pandemic
Source: Eur J Public Health. 2023 Jul 3;33(5):844–50. doi: 10.1093/eurpub/ckad104 (PMC10567130; doi:10.1093/eurpub/ckad104)
Supplement: ckad104_Supplementary_Data [file ckad104_supplementary_data.zip › ckad104_Supplementary_Data/ejph-2023-02-om-0064-File003.docx]

**Supplementary Appendix for the article:**

**Changes in tobacco and nicotine use in Finland during the COVID-19 pandemic**

Sebastián Peña, Katja Ilmarinen, Laura Kestilä, Otto Ruokolainen, Hanna Ollila, Suvi Parikka, Sakari Karvonen

**Correspondence:**

Sebastián Peña

Finnish Institute for Health and Welfare

Mannerheimintie 166

Helsinki

FINLAND

Phone: +358 45 245 1360

Email: sebastian.penafajuri@thl.fi

**Table of Contents**

[1. Changes in design, definitions and methods compared to registered protocol 3](#_7wqhtsq69v1s)

[2. Harmonization of FinSote survey questions and construction of outcomes and covariates 4](#_vol9hgd3scdm)

[2.1 Outcomes 4](#_r041g18m5lms)

[2.2 Sociodemographic factors 8](#_670xmtem71yz)

[3. Differences in survey questionnaires for smoking outcomes 11](#_p5vd9jvq6xn1)

[4. Description of inverse probability weights 12](#_h3zgbqu71vvp)

[5. Missing data in outcomes and sociodemographic factors in FinSote surveys, 2018-2020 13](#_8uk6i6ty2o4d)

[6. Results of interaction tests 15](#_m4c2vlef7il1)

[7. Table S3. Model-adjusted prevalences and 95% confidence intervals of smoking among 38675 participants aged 20 to 74 years old by sex, 2018-2020 17](#_z4yi69kwoyul)

[8. Model-adjusted prevalences of occasional tobacco or nicotine use by subgroups 18](#_v00h484814n6)

[9. Table S4. Model-adjusted prevalences of smoking status by sociodemographic characteristics, 2018-2020 20](#_v5ikk2ipr4eb)

[10. Table S5. Model-adjusted prevalences of snus status by sociodemographic characteristics, 2018-2020 22](#_tbj2wx4kxd0a)

[11. Table S6. Model-adjusted prevalences of e-cigarettes with nicotine by sociodemographic characteristics, 2018-2020 24](#_lv3473grz4p7)

[12. Table S7. Model-adjusted prevalences of e-cigarettes without nicotine by sociodemographic characteristics, 2018-2020 26](#_ls0gefl3rixt)

[13. Table S8. Model-adjusted prevalences of NRT products by sociodemographic characteristics, 2018-2020 28](#_9g0dot383eq9)

[14. Table S9. Model-adjusted prevalences of any daily tobacco or nicotine use by sociodemographic characteristics, 2018-2020 30](#_zeh826welwah)

Statistical code in R and Stata are provided as additional files.

# Changes in design, definitions and methods compared to registered protocol

- The study design was identical to the pre-registered protocol
- There were minor changes to definitions. We categorised age into three categories and not in six categories because the interaction models between age and survey year ran into singular fit problems due to the data being too sparse in some categories. We did not report prevalence by hospital district given the data was too sparse in some areas. We defined total use of tobacco or nicotine products as the current daily use (the protocol states “current use”) of tobacco, snus, e-cigarettes with nicotine and NRT products to keep consistency with the goals of a Tobacco-free Finland 2030 and, therefore, make it more relevant for national policy-makers.
- There were minor changes to the outcomes. The protocol stated that the primary outcomes were the prevalence of smoking, snus use, e-cigarettes use and NRT products and the secondary outcome was total tobacco or nicotine use. We changed NRT to a secondary outcome because it is a medicinal product and, therefore, belonged to a different category than the other outcomes. We defined the total tobacco and nicotine use as a primary outcome given the importance for Finnish policy goals.
- There were minor changes in the methods. We decided not to report odds ratios as the study is descriptive in nature and we are not reporting fully-adjusted estimates to examine a causal association. We, instead, report model-adjusted prevalences and prevalence differences between years 2018 to 2020.

#

#

#

# Harmonization of FinSote survey questions and construction of outcomes and covariates

We harmonised the three FinSote surveys using a structured protocol. The original forms in Finnish, Swedish, English and Russian are available [here](https://thl.fi/fi/tutkimus-ja-kehittaminen/tutkimukset-ja-hankkeet/finsote-tutkimus/miten-finsote-tutkimus-tehdaan/lomakkeet).

## ***2.1 Outcomes***

2.1.1 Smoking status

Variable: smoking_status

Type: Factor, [“never smoker”, “former smoker”, “current occasional smoker”, “current daily smoker”]

Description. Smoking was asked in FinSote 2018 and 2020 with the question “Do you smoke currently (cigarettes, cigars or pipe)?”. FinSote 2019 was implemented together with EHIS 3 and thus questions were different but still very comparable. The first question asked “Do you smoke at present (other tobacco products than e-cigarettes)?” which allowed us to separate daily and occasional smokers. If the answer was “not at all”, respondents would be directed to another question “Have you ever smoked daily for a period of at least one year? For how many years altogether?” which allows us to separate former and never smokers.

Questions, answers and equivalence are shown below

| **Survey** | **Question** | **Answer** | **Equivalence** |
| --- | --- | --- | --- |
| FinSote 2018 | 84. Do you smoke currently (cigarettes, cigars or pipe)? | 1.yes, daily  2. occasionally  3. not at all  4. I have never smoked | Current daily smoker 1  Current occasional smoker 2  Former smoker 3  Never smoker 4 |
| FinSote 2019 | 73. Do you smoke at present (other tobacco products than e-cigarettes)?  76. Have you ever smoked daily for a period of at least one year? For how many years altogether? | 1. yes, daily  2. Yes, occasionally  3. not at all  1. I have never smoked daily  2. I have smoked daily for a total of _ years | Current daily smoker Q73=1  Current occasional smoker Q73=2  Former smoker Q73=3 & Q76=2  Never smoker Q73=3 & Q76=1 |
| FinSote 2020 | 62. Do you smoke currently (cigarettes, cigars or pipe)? | 1.yes, daily  2. occasionally  3. not at all  4. I have never smoked | Current daily smoker 1  Current occasional smoker 2  Former smoker 3  Never smoker 4 |

2.1.2 Snus status

Variable: snus_status

Type: Factor, [“never user”, “former user”, “current user”]

Description. Snus use was asked in FinSote 2018 and 2020 with a question “Do you currently use any of the following products? snus (Swedish type moist snuff)” and in FinSote 2019 as “Do you currently use snuff?”. This question was not available in FinSote 2018 and 2020 in the questionnaire for 75+ as it was assumed snus use in this age category is very uncommon.

Questions, answers and equivalence are shown below:

| **Survey** | **Question** | **Answer** | **Equivalence** |
| --- | --- | --- | --- |
| FinSote 2018 | 85a. Do you currently use any of the following products?  snus (Swedish type moist snuff) | 1. yes, daily  2. occasionally  3. not at all  4. I have never used | Current user 1 or 2  Former user 3  Never user 4 |
| FinSote 2019 | 78. Do you currently use snuff?  76. Have you ever smoked daily for a period of at least one year? For how many years altogether? | 1. yes, daily  2. yes, occasionally  3. not at all  4. I have never used it | Current user 1 or 2  Former user 3  Never user 4 |
| FinSote 2020 | 63a. Do you currently use any of the following products?  snus (Swedish type moist snuff) | 1. yes, daily  2. occasionally  3. not at all  4. I have never used | Current user 1 or 2  Former user 3  Never user 4 |

2.1.3 E-cigarettes with nicotine

Variable: ecig_nic_status

Type: Factor, [“never user”, “former user”, “current user”]

Description. The use of e-cigarettes with nicotine was asked in FinSote 2018 and 2020 with a question “Do you currently use any of the following products? e-cigarettes with nicotine”. FinSote includes a question on general use of e-cigarettes “Do you currently use electronic cigarettes (e-cigarettes)?” but we considered it necessary to separate whether they contained nicotine as it is a crucial component of the research question. This question was not available in FinSote 2018 and 2020 in the questionnaire for 75+ as it was assumed e-cigarette use in this age category was very uncommon.

Questions, answers and equivalence are shown below:

| **Survey** | **Question** | **Answer** | **Equivalence** |
| --- | --- | --- | --- |
| FinSote 2018 | 85b. Do you currently use any of the following products?  e-cigarettes with nicotine | 1. yes, daily  2. occasionally  3. not at all  4. I have never used | Current user 1 or 2  Former user 3  Never user 4 |
| FinSote 2020 | 63b. Do you currently use any of the following products?  e-cigarettes with nicotine | 1. yes, daily  2. occasionally  3. not at all  4. I have never used | Current user 1 or 2  Former user 3  Never user 4 |

2.1.4 E-cigarettes without nicotine

Variable: ecig_nonic_status

Type: Factor, [“never user”, “former user”, “current user”]

Description. The use of e-cigarettes without nicotine was asked in FinSote 2018 and 2020 with a question “Do you currently use any of the following products? e-cigarettes without nicotine”. This question was not available in FinSote 2018 and 2020 in the questionnaire for 75+ as it was assumed e-cigarette use in this age category was very uncommon.

Questions, answers and equivalence are shown below:

| **Survey** | **Question** | **Answer** | **Equivalence** |
| --- | --- | --- | --- |
| FinSote 2018 | 85c. Do you currently use any of the following products?  e-cigarettes without nicotine | 1. yes, daily  2. occasionally  3. not at all  4. I have never used | Current user 1 or 2  Former user 3  Never user 4 |
| FinSote 2020 | 63c. Do you currently use any of the following products?  e-cigarettes without nicotine | 1. yes, daily  2. occasionally  3. not at all  4. I have never used | Current user 1 or 2  Former user 3  Never user 4 |

2.1.5 Nicotine replacement therapy products

Variable: nrt_status

Type: Factor, [“never user”, “former user”, “current user”]

Description. The use of nicotine replacement products was asked in FinSote 2018 and 2020 with a question “Do you currently use any of the following products? nicotine replacement therapy products such as patches or chewing gum”. This question was not available in FinSote 2018 and 2020 in the questionnaire for 75+.

Questions, answers and equivalence are shown below:

| **Survey** | **Question** | **Answer** | **Equivalence** |
| --- | --- | --- | --- |
| FinSote 2018 | 85d. Do you currently use any of the following products?  nicotine replacement therapy products such as patches or chewing gum | 1. yes, daily  2. occasionally  3. not at all  4. I have never used | Current user 1 or 2  Former user 3  Never user 4 |
| FinSote 2020 | 63d. Do you currently use any of the following products?  nicotine replacement therapy products such as patches or chewing gum | 1. yes, daily  2. occasionally  3. not at all  4. I have never used | Current user 1 or 2  Former user 3  Never user 4 |

## ***2.2 Sociodemographic factors***

2.2.1 Sex

Variable: sex

Type: Binary, [male=0, female=1]

Description. We obtained information on participants’ sex from registries administered by the Digital and Population Data Services Agency.

2.2.2 Age

Variable: age_cont

Type: Continuous, [natural number]

Description. We obtained information on participants’ age from registries administered by the Digital and Population Data Services Agency.

2.2.4 Years of education

Variable:educ_years

Type: Continuous, [natural number]

Description. All three surveys had identical questions on the number of years of full time studies.

Questions, answers and equivalence are shown below:

| **Survey** | **Question** | **Answer** |
| --- | --- | --- |
| FinSote 2018 | 2. How many years altogether have you attended school or studied full time? Including primary and comprehensive school. | _ years |
| FinSote 2019 | 4. How many years altogether have you attended school or studied full time? Including primary and comprehensive school. | _ years |
| FinSote 2020 | 4. How many years altogether have you attended school or studied full time? Including primary and comprehensive school. | _ years |

2.2.3 Marital status

Variable: marital_status

Type: Factor, [“married, in a registered relationship or cohabiting”, “separated, divorced, widowed or single”]

Description. All surveys had an identical question on marital status. Responders were asked “Are you currently: married or in a registered relationship, cohabiting, separated or divorced, widowed, single”. We created a categorical variable with two categories.

Questions, answers and equivalence are shown below:

| **Survey** | **Question** | **Answer** | **Equivalence** |
| --- | --- | --- | --- |
| FinSote 2018 | 1. Are you currently: | 1. Married or in a registered relationship  2. cohabiting  3. separated or divorced  4. widowed  5. single | Married, in a registered relationship or cohabiting 1 or 2  Separated, divorced, widowed or single 3, 4 or 5 |
| FinSote 2019 | 1. Are you currently: | 1. Married or in a registered relationship  2. cohabiting  3. separated or divorced  4. widowed  5. single | Married, in a registered relationship or cohabiting 1 or 2  Separated, divorced, widowed or single 3, 4 or 5 |
| FinSote 2020 | 3. Are you currently: | 1. Married or in a registered relationship  2. cohabiting  3. separated or divorced  4. widowed  5. single | Married, in a registered relationship or cohabiting 1 or 2  Separated, divorced, widowed or single 3, 4 or 5 |

2.2.5 Mother tongue

Variable: mother_tongue

Type: Factor, [“Finnish”, “Swedish” and “others”]

Description. We obtained information on participants’ mother tongue from registries administered by the Digital and Population Data Services Agency.

2.2.7 Participation in social activities

Variable: involvement_attend_j

Type: Factor, [“no participation”, “occasional”, “active”]

Description. All three surveys had an identical question on participation in social activities.

Questions, answers and equivalence are shown below:

| **Survey** | **Question** | **Answer** | **Equivalence** |
| --- | --- | --- | --- |
| FinSote 2018 | 11. Do you participate in the activities of any club, association, hobby group or religious or spiritual community (e.g. a sports club, residents’ association, political party, choir, parish)? | 1.no  2. yes, actively  3. Yes, occasionally | No participation 1  Occasional 3  Active 2 |
| FinSote 2019 | 89. Do you participate in the activities of any club, association, hobby group or religious or spiritual community (e.g. a sports club, residents’ association, political party, choir, parish)? | 1.no  2. yes, actively  3. Yes, occasionally | No participation 1  Occasional 3  Active 2 |
| FinSote 2020 | 12. Do you participate in the activities of any club, association, hobby group or religious or spiritual community (e.g. a sports club, residents’ association, political party, choir, parish)? | 1.no  2. yes, actively  3. Yes, occasionally | No participation 1  Occasional 3  Active 2 |

#

# Differences in survey questionnaires for smoking outcomes

The question assessing smoking in 2018 and 2020 was “Do you smoke currently (cigarettes, cigars or pipe)?” from which current daily, occasional, former and never users could be directly derived. The question in 2019 consisted of two parts. The first question was “Do you smoke at present (other tobacco products than e-cigarettes)?” with possible answers (yes, daily)(yes, occasionally)(not at all). Those who answered “not at all” were asked a second question “Have you ever smoked daily for a period of at least one year? For how many years altogether?” with two possible answers: (I have never smoked daily)(I have smoked daily for a total of ___________ years). This question allows to separate never **daily** smokers from former **daily** smokers, which is not the same as the questions in 2018 and 2020. For this reason, the proportions of never and former smokers were very comparable for the years 2018 and 2020, but not for 2019. However, as the questions for current daily and occasional smokers are almost identical, collapsing the category into never **and** former smokers creates very similar proportions for all years.

#

# Description of inverse probability weights

FinSote surveys used similar inverse probability weights for each survey year. These weights are used to provide representative information of the Finnish population aged 20 years and older but also to correct for unit non-response (non-participation). The weighting process used data from national registries available for the entire sample, including age, sex, marital status, level of education, mother tongue and area of residence. The probability of response is modeled for each respondent, and a weighting factor for each subject is calculated as the inverse of this probability and the sampling probability. This method has been validated using health survey data in Finland, see Härkänen et al (2014) for details.[^1^](https://www.zotero.org/google-docs/?Sn4mzl)

[1. Härkänen, T., Kaikkonen, R., Virtala, E. & Koskinen, S. Inverse probability weighting and doubly robust methods in correcting the effects of non-response in the reimbursed medication and self-reported turnout estimates in the ATH survey. *BMC Public Health* **14**, 1150 (2014).](https://www.zotero.org/google-docs/?fZAS7a)

#

# Missing data in outcomes and sociodemographic factors in FinSote surveys, 2018-2020

We had complete data for sex, age and mother tongue, as this data comes from national registries. Missing data for the outcomes were fairly consistent across study years, although there is a small tendency for FinSote 2020 to have lower missing data. This might be related to health awareness during the COVID-19 pandemic. Socioeconomic factors were also consistent across study years, with the exception of education which had a higher percentage of missing data in 2018. However, this remained below 10% and, therefore, differences in non-response between educational groups would need to be very large to influence our results.

**Table S1**. Missing data in outcomes and sociodemographic factors by FinSote survey wave

|  | **2018** | | | **2019** | | | **2020** | | |
| --- | --- | --- | --- | --- | --- | --- | --- | --- | --- |
| **Variable** | **Survey sample** | **Complete data** | **Missing, %** | **Survey sample** | **Complete data** | **Missing, %** | **Survey sample** | **Complete data** | **Missing, %** |
| *Age range 20 and older* |  |  |  |  |  |  |  |  |  |
| Smoking status | 26422 | 25451 | 3.7 | 5943 | 5675 | 4.5 | 28199 | 27400 | 2.8 |
| Sex | 26422 | 26422 | 0 | 5943 | 5943 | 0 | 28199 | 28199 | 0.0 |
| Age | 26422 | 26422 | 0 | 5943 | 5943 | 0 | 28199 | 28199 | 0.0 |
| Educational level | 26422 | 24265 | 8.2 | 5943 | 5748 | 3.3 | 28199 | 26896 | 4.6 |
| Marital status | 26422 | 25830 | 2.2 | 5943 | 5881 | 1.0 | 28199 | 27763 | 1.5 |
| Mother tongue | 26422 | 26422 | 0 | 5943 | 5943 | 0 | 28199 | 28199 | 0.0 |
| Participation in social activities | 26422 | 25889 | 2.0 | 5943 | 5839 | 1.7 | 28199 | 27679 | 1.8 |
|  |  |  |  |  |  |  |  |  |  |
| *Age range 20 to 74 years old* |  |  |  |  |  |  |  |  |  |
| Smoking status | 16641 | 16036 | 3.6 | 5051 | 4857 | 3.8 | 18280 | 17782 | 2.7 |
| Snus status | 16641 | 16035 | 3.6 | 5051 | 4971 | 1.6 | 18280 | 17748 | 2.9 |
| E-cigarretes with nicotine | 16641 | 16004 | 3.8 | 5051 | 0 | 100 | 18280 | 17706 | 3.1 |
| E-cigarettes without nicotine | 16641 | 15933 | 4.3 | 5051 | 0 | 100 | 18280 | 17629 | 3.6 |
| NRT products | 16641 | 16008 | 3.8 | 5051 | 0 | 100 | 18280 | 17672 | 3.3 |
| Any tobacco or nicotine product | 16641 | 16227 | 2.5 | 5051 | 0 | 100 | 18280 | 17985 | 1.6 |
| Sex | 16641 | 16641 | 0 | 5051 | 5051 | 0 | 18280 | 18280 | 0 |
| Age | 16641 | 16641 | 0 | 5051 | 5051 | 0 | 18280 | 18280 | 0 |
| Educational level | 16641 | 15957 | 4.1 | 5051 | 4932 | 2.4 | 18280 | 17844 | 2.4 |
| Marital status | 16641 | 16389 | 1.5 | 5051 | 5003 | 1.0 | 18280 | 18140 | 0.8 |
| Mother tongue | 16641 | 16641 | 0 | 5051 | 5051 | 0 | 18280 | 18280 | 0 |
| Participation in social activities | 16641 | 16418 | 1.3 | 5051 | 4977 | 1.5 | 18280 | 18105 | 1.0 |

Data are counts and percentages (%).

# Results of interaction tests

In Table S2, we report the results of interaction coefficients between the outcome and survey year and the p-values for all interaction models. We found evidence of an interaction between survey year and smoking (i.e. prevalence of smoking decreased linearly in the study period) and between age and snus use and age and use of nicotine replacement therapy (NRT) products.

Some models had too sparse data (i.e. due to the low prevalence of the outcome or sparsely populated categories), resulting in singular fit errors in the models. In those cases, it was not possible to obtain a p-value for the interaction term.

**Table S2**. Models and estimates of interaction tests

| **Model** | **Interaction coefficient** | **p-value** |
| --- | --- | --- |
| *Interactions over time* |  |  |
| Smoking + survey year | -0.049 | **0.020** |
| Snus use + survey year | -0.001 | 0.976 |
| E-cigarettes with nicotine + survey year | -0.008 | 0.756 |
| E-cigarettes without nicotine + survey year | -0.007 | 0.802 |
| Any daily tobacco or nicotine + survey year | -0.036 | 0.145 |
| NRT products + survey year | 0.013 | 0.553 |
|  |  |  |
| *Interactions by subgroups - Smoking* |  |  |
| + age + sex*survey year |  | 0.555 |
| + sex + age*survey year |  | 0.981 |
| + sex + age + education*survey year |  | 0.482 |
| + sex + age + marital status*survey year |  | 0.464 |
| + sex + age + mother tongue*survey year |  | 0.666 |
| + sex + age + social participation*survey year |  | 0.412 |
|  |  |  |
| *Interactions by subgroups - Snus use* |  |  |
| + age + sex*survey year |  | 0.463 |
| + sex + age*survey year |  | **2.36E-06** |
| + sex + age + education*survey year |  | Singular fit |
| + sex + age + marital status*survey year |  | 0.864 |
| + sex + age + mother tongue*survey year |  | 0.698 |
| + sex + age + social participation*survey year |  | 0.221 |
|  |  |  |
| *Interactions by subgroups - E-cigarettes with nicotine* | | |
| + age + sex*survey year |  | 0.269 |
| + sex + age*survey year |  | Singular fit |
| + sex + age + education*survey year |  | 0.766 |
| + sex + age + marital status*survey year |  | Singular fit |
| + sex + age + mother tongue*survey year |  | Singular fit |
| + sex + age + social participation*survey year |  | Singular fit |
|  |  |  |
| *Interactions by subgroups - E-cigarettes without nicotine* | | |
| + age + sex*survey year |  | Singular fit |
| + sex + age*survey year |  | Singular fit |
| + sex + age + education*survey year |  | Singular fit |
| + sex + age + marital status*survey year |  | Singular fit |
| + sex + age + mother tongue*survey year |  | 0.724 |
| + sex + age + social participation*survey year |  | Singular fit |
|  |  |  |
| *Interactions by subgroups - Any daily tobacco or nicotine use* | | |
| + age + sex*survey year |  | 0.589 |
| + sex + age*survey year |  | 0.455 |
| + sex + age + education*survey year |  | 0.837 |
| + sex + age + marital status*survey year |  | 0.531 |
| + sex + age + mother tongue*survey year |  | 0.717 |
| + sex + age + social participation*survey year |  | 0.371 |
|  |  |  |
| *Interactions by subgroups - NRT products* |  |  |
| + age + sex*survey year |  | 0.174 |
| + sex + age*survey year |  | **0.00137** |
| + sex + age + education*survey year |  | Singular fit |
| + sex + age + marital status*survey year |  | Singular fit |
| + sex + age + mother tongue*survey year |  | 0.763 |
| + sex + age + social participation*survey year |  | Singular fit |

NRT nicotine replacement therapy. Models are ordered logistic regressions which incorporate sampling design. Survey year was modelled as a continuous variable. P-values were obtained from adjusted Wald tests using the *testparm* command in Stata.

#

# Table S3. Model-adjusted prevalences and 95% confidence intervals of smoking among 38675 participants aged 20 to 74 years old by sex, 2018-2020

#

|  | **Males** | | | **Females** | | |
| --- | --- | --- | --- | --- | --- | --- |
|  | **2018** | **2019** | **2020** | **2018** | **2019** | **2020** |
| Daily | 15.0 (13.9; 16.1) | 14.3 (13.1; 15.4) | 13.7 (12.9; 14.5) | 11.1 (10.3; 11.9) | 10.5 (9.6; 11.5) | 10.1 (9.4; 10.7) |
| Occasional | 9.0 (8.4; 9.7) | 8.7 (8; 9.4) | 8.4 (7.9; 9) | 7.2 (6.7; 7.7) | 6.9 (6.3; 7.5) | 6.7 (6.2; 7.1) |
| Never smoker | 76.0 (74.5; 77.5) | 77.0 (75.4; 78.6) | 77.9 (76.8; 79.0) | 81.7 (80.5; 82.9) | 82.6 (81.3; 83.9) | 83.3 (82.4; 84.2) |

Data are percentages (95% CI). Prevalence estimates are age-adjusted and obtained using 2020 as the reference population. All prevalences incorporate the complex sampling design and represent the Finnish population. Data on smoking was available for the whole sample (aged 20 and over). Data on snus, e-cigarettes with or without nicotine and NRT products was available for those aged 20-74 years old.

# Model-adjusted prevalences of occasional tobacco or nicotine use by subgroups

**Figure S1**. Model-adjusted prevalences of occasional tobacco and nicotine use in Finland by age, educational tertiles, marital status, mother tongue and participation in social activities, 2018-2020


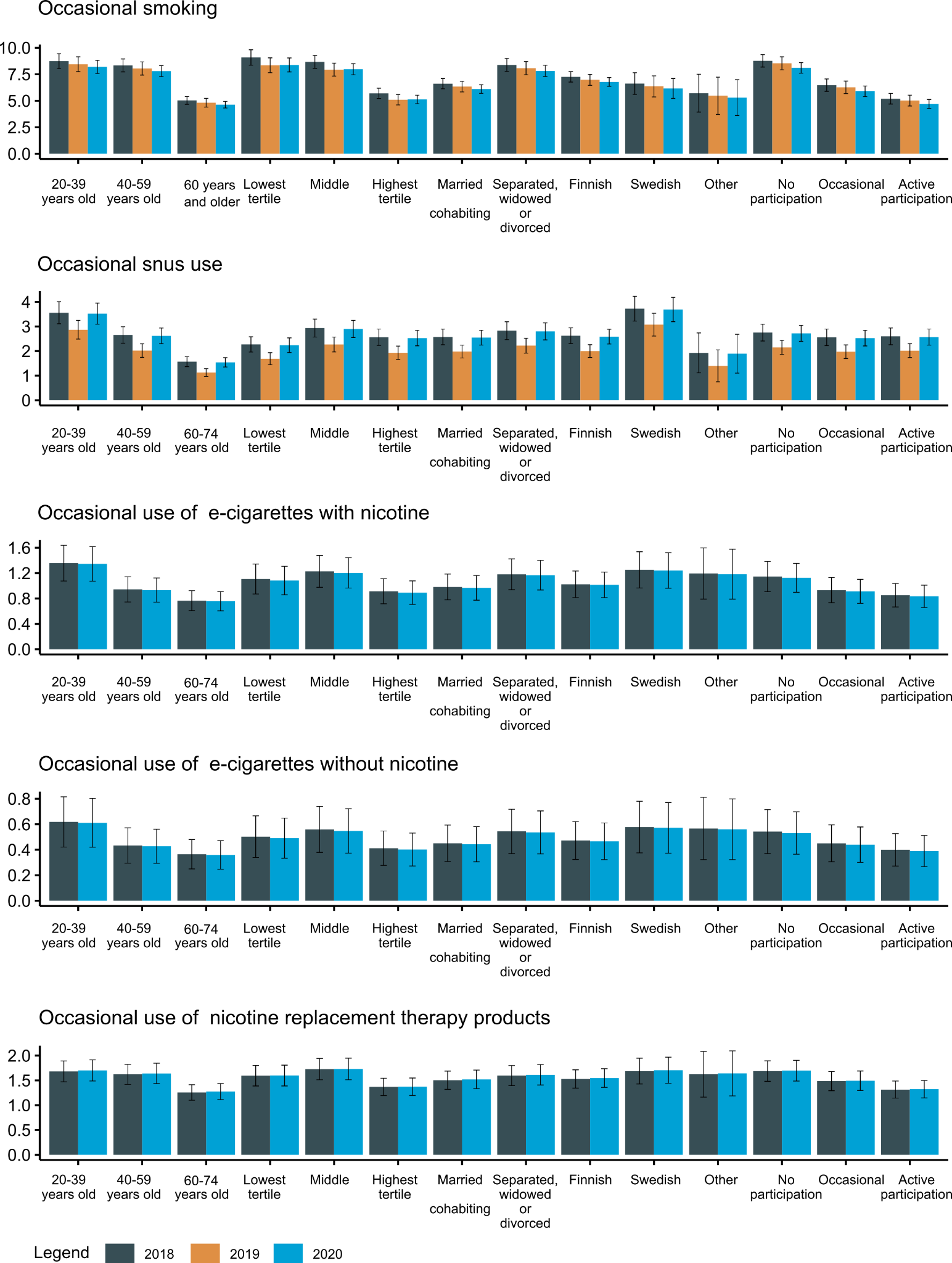


Note: Estimates are predicted means from ordered logistic regression models. Data on smoking was available for those aged 20 years and older. Data on snus, e-cigarettes with or without nicotine and NRT products was available for those 20-74 years old.

# **Table S4**. Model-adjusted prevalences of smoking status by sociodemographic characteristics, 2018-2020

|  | **2018** | **2019** | **2020** |
| --- | --- | --- | --- |
| **Age** |  |  |  |
| *20-39 years old* |  |  |  |
| Daily smoker | 14.8 (13.6; 16) | 14.2 (13; 15.3) | 13.6 (12.7; 14.5) |
| Occasional smoker | 8.7 (8; 9.4) | 8.4 (7.7; 9.1) | 8.2 (7.6; 8.8) |
| Never or former smoker | 76.4 (74.8; 78.1) | 77.4 (75.7; 79.1) | 78.2 (76.9; 79.5) |
| *40-59 years old* |  |  |  |
| Daily smoker | 13.9 (12.8; 15) | 13.3 (12.1; 14.4) | 12.7 (11.9; 13.6) |
| Occasional smoker | 8.3 (7.7; 8.9) | 8 (7.4; 8.7) | 7.8 (7.3; 8.3) |
| Never or former smoker | 77.8 (76.3; 79.3) | 78.7 (77.1; 80.3) | 79.5 (78.3; 80.6) |
| *60 years and older* |  |  |  |
| Daily smoker | 7.4 (6.9; 8) | 7.1 (6.4; 7.7) | 6.8 (6.3; 7.2) |
| Occasional smoker | 5 (4.7; 5.4) | 4.8 (4.4; 5.2) | 4.6 (4.3; 4.9) |
| Never or former smoker | 87.5 (86.7; 88.3) | 88.1 (87.1; 89.1) | 88.6 (88; 89.3) |
| **Educational level** |  |  |  |
| *Lower tertile* |  |  |  |
| Daily smoker | 15.7 (14.2; 17.3) | 14. (12.6; 15.3) | 14. (12.7; 15.3) |
| Occasional smoker | 9.1 (8.3; 9.8) | 8.3 (7.6; 9) | 8.4 (7.7; 9) |
| Never or former smoker | 75.2 (73.1; 77.2) | 77.7 (75.8; 79.6) | 77.6 (75.8; 79.4) |
| *Middle tertile* |  |  |  |
| Daily smoker | 14.7 (13.6; 15.8) | 13 (11.9; 14.1) | 13.1 (12.3; 13.9) |
| Occasional smoker | 8.7 (8.1; 9.3) | 7.9 (7.3; 8.6) | 8 (7.4; 8.5) |
| Never or former smoker | 76.6 (75.2; 78.1) | 79 (77.5; 80.5) | 78.9 (77.9; 80) |
| *Highest tertile* |  |  |  |
| Daily smoker | 8.5 (7.8; 9.2) | 7.4 (6.7; 8.2) | 7.5 (7; 8) |
| Occasional smoker | 5.7 (5.2; 6.2) | 5.1 (4.6; 5.6) | 5.1 (4.7; 5.5) |
| Never or former smoker | 85.8 (84.7; 86.9) | 8 (7.4; 8.5) | 14.7 (13.6; 15.8) |
| **Marital status** |  |  |  |
| *Married or cohabiting* |  |  |  |
| Daily smoker | 10.4 (9.7; 11.2) | 9.9 (9.1; 10.7) | 9.4 (8.9; 10) |
| Occasional smoker | 6.6 (6.1; 7.1) | 6.3 (5.8; 6.8) | 6.1 (5.7; 6.5) |
| Never or former smoker | 82.9 (81.9; 84) | 87.4 (86.3; 88.6) | 87.4 (86.6; 88.2) |
| *Separated, widowed or divorced* | | | |
| Daily smoker | 14.3 (13.2; 15.4) | 13.6 (12.4; 14.7) | 13 (12.1; 13.8) |
| Occasional smoker | 8.4 (7.8; 9) | 8.1 (7.4; 8.7) | 7.8 (7.3; 8.3) |
| Never or former smoker | 77.3 (75.9; 78.8) | 78.4 (76.8; 79.9) | 79.2 (78.1; 80.3) |
| **Mother tongue** |  |  |  |
| *Finnish* |  |  |  |
| Daily smoker | 11.9 (11.1; 12.6) | 11.3 (10.4; 12.1) | 10.8 (10.3; 11.4) |
| Occasional smoker | 7.3 (6.8; 7.7) | 7 (6.5; 7.5) | 6.8 (6.4; 7.2) |
| Never or former smoker | 80.9 (79.9; 81.9) | 81.8 (80.6; 82.9) | 82.4 (81.7; 83.1) |
| *Swedish* |  |  |  |
| Daily smoker | 10.5 (8.6; 12.5) | 10 (8.1; 11.9) | 9.6 (5.6; 7.3) |
| Occasional smoker | 6.2 (5.2; 7.1) | 82.8 (79.9; 85.7) | 83.6 (80.8; 86.4) |
| Never or former smoker | 84.2 (81.6; 86.8) | 8.8 (5.5; 12) | 8.3 (5.2; 11.5) |
| *Other* |  |  |  |
| Daily smoker | 8.8 (5.5; 12) | 8.3 (5.2; 11.5) | 8 (5; 11) |
| Occasional smoker | 5.7 (3.9; 7.5) | 5.5 (3.7; 7.2) | 5.3 (3.6; 7) |
| Never or former smoker | 85.5 (80.5; 90.5) | 6.8 (6.4; 7.2) | 11.9 (11.1; 12.6) |
| **Participation in social activities** | | | |
| *Active participation* |  |  |  |
| Daily smoker | 7.6 (6.9; 8.4) | 7.3 (6.5; 8.1) | 6.8 (6.2; 7.4) |
| Occasional smoker | 5.2 (4.7; 5.7) | 5 (4.5; 5.5) | 4.7 (4.3; 5.1) |
| Never or former smoker | 87.2 (86; 88.3) | 87.7 (86.5; 88.9) | 88.5 (87.6; 89.5) |
| *Occasional* |  |  |  |
| Daily smoker | 10 (9; 10.9) | 9.6 (8.6; 10.5) | 8.9 (8.1; 9.6) |
| Occasional smoker | 6.5 (5.9; 7.1) | 6.3 (5.7; 6.9) | 5.9 (5.4; 6.4) |
| Never or former smoker | 83.6 (82.2; 84.9) | 84.2 (82.7; 85.6) | 6.5 (5.9; 7.1) |
| *No participation* |  |  |  |
| Daily smoker | 14.9 (13.9; 15.9) | 14.3 (13.2; 15.5) | 13.3 (12.6; 14.1) |
| Occasional smoker | 8.8 (8.2; 9.3) | 8.5 (7.9; 9.1) | 8.1 (7.6; 8.6) |
| Never or former smoker | 76.4 (75; 77.7) | 13.3 (12.6; 14.1) | 84.2 (82.7; 85.6) |

# **Table S5**. Model-adjusted prevalences of snus status by sociodemographic characteristics, 2018-2020

|  | **2018** | **2019** | **2020** |
| --- | --- | --- | --- |
| **Age** |  |  |  |
| *20-39 years old* |  |  |  |
| Daily user | 3.3 (2.9; 3.7) | 2.9 (2.5; 3.3) | 3.4 (3; 3.8) |
| Occasional user | 3.4 (3; 3.9) | 3 (2.5; 3.4) | 3.6 (3.1; 4) |
| Former user | 19.1 (17.2; 21) | 15.4 (13.6; 17.2) | 20.1 (18.9; 21.4) |
| Never user | 74.2 (71.9; 76.5) | 78.8 (76.6; 81) | 73 (71.4; 74.5) |
| *40-59 years old* |  |  |  |
| Daily user | 2.5 (2.2; 2.9) | 2.1 (1.7; 2.4) | 2.6 (2.3; 2.9) |
| Occasional user | 2.6 (2.2; 3) | 2.1 (1.7; 2.4) | 2.7 (2.4; 3) |
| Former user | 12.9 (11.5; 14.3) | 9.7 (8.4; 11.1) | 13.5 (12.5; 14.6) |
| Never user | 81.9 (80.1; 83.8) | 86.2 (84.4; 87.9) | 81.2 (79.9; 82.4) |
| *60 to 74 years old* |  |  |  |
| Daily user | 1.9 (1.6; 2.1) | 1 (0.8; 1.2) | 1.4 (1.2; 1.6) |
| Occasional user | 1.9 (1.6; 2.1) | 1 (0.8; 1.2) | 1.4 (1.2; 1.5) |
| Former user | 8.7 (7.8; 9.7) | 4.3 (3.5; 5) | 6 (5.4; 6.6) |
| Never user | 87.5 (86.3; 88.7) | 93.7 (92.6; 94.8) | 91.2 (90.5; 92) |
| **Educational level** |  |  |  |
| *Lower tertile* |  |  |  |
| Daily user | 2.8 (2.5; 3.2) | 2.2 (1.9; 2.5) | 2.8 (2.4; 3.1) |
| Occasional user | 2.9 (2.5; 3.3) | 2.2 (1.9; 2.6) | 2.8 (2.5; 3.2) |
| Former user | 15.4 (13.7; 17) | 11.1 (9.8; 12.4) | 15.2 (13.7; 16.7) |
| Never user | 78.9 (76.8; 80.9) | 84.4 (82.8; 86.1) | 79.2 (77.4; 81.1) |
| *Middle tertile* |  |  |  |
| Daily user | 2.8 (2.4; 3.1) | 2.2 (1.9; 2.4) | 2.7 (2.4; 3) |
| Occasional user | 2.8 (2.5; 3.2) | 2.2 (1.9; 2.4) | 2.7 (2.4; 3.1) |
| Former user | 14.7 (13.5; 15.9) | 10.6 (9.6; 11.6) | 14.5 (13.6; 15.4) |
| Never user | 79.7 (78.3; 81.2) | 85.1 (83.8; 86.4) | 80.1 (79; 81.2) |
| *Highest tertile* |  |  |  |
| Daily user | 2.5 (2.2; 2.8) | 1.9 (1.7; 2.2) | 2.4 (2.2; 2.7) |
| Occasional user | 2.5 (2.2; 2.9) | 1.9 (1.6; 2.2) | 2.5 (2.2; 2.8) |
| Former user | 12.8 (11.7; 13.8) | 9.2 (8.2; 10.1) | 12.6 (11.8; 13.4) |
| Never user | 82.2 (80.9; 83.5) | 87 (85.8; 88.2) | 82.5 (81.4; 83.5) |
| **Marital status** |  |  |  |
| *Married or cohabiting* | | | |
| Daily user | 2.6 (2.3; 2.9) | 2 (1.8; 2.3) | 2.5 (2.2; 2.8) |
| Occasional user | 2.7 (2.4; 3) | 2.1 (1.8; 2.3) | 2.6 (2.3; 2.9) |
| Former user | 13.9 (12.9; 14.9) | 10.1 (9.2; 11) | 13.7 (13; 14.5) |
| Never user | 80.8 (79.6; 82.1) | 85.7 (84.6; 86.9) | 81.1 (80.2; 82) |
| *Separated, widowed or divorced* | | | |
| *Daily user* | 2.6 (2.3; 2.9) | 2 (1.8; 2.3) | 2.5 (2.2; 2.8) |
| Occasional user | 2.7 (2.4; 3) | 2.1 (1.8; 2.4) | 2.6 (2.3; 3) |
| Former user | 14. (12.8; 15.1) | 10.2 (9.2; 11.2) | 13.8 (12.8; 14.8) |
| Never user | 80.7 (79.3; 82.2) | 85.7 (84.4; 87) | 81 (79.9; 82.2) |
| **Mother tongue** |  |  |  |
| *Finnish* |  |  |  |
| Daily user | 2.6 (2.3; 2.9) | 2 (1.8; 2.3) | 2.5 (2.2; 2.8) |
| Occasional user | 2.7 (2.3; 3) | 2 (1.8; 2.3) | 2.6 (2.3; 2.9) |
| Former user | 13.7 (12.7; 14.6) | 9.8 (9; 10.7) | 13.4 (12.8; 14.1) |
| Never user | 81.1 (80; 82.2) | 86.1 (85.1; 87.2) | 81.5 (80.7; 82.3) |
| *Swedish* |  |  |  |
| Daily user | 3.5 (3.1; 4) | 2.9 (2.5; 3.3) | 3.4 (3; 3.8) |
| Occasional user | 3.8 (3.3; 4.3) | 3 (2.6; 3.5) | 3.6 (3.2; 4.1) |
| Former user | 22.9 (20.2; 25.5) | 17 (14.8; 19.2) | 22.4 (19.9; 24.8) |
| Never user | 69.9 (66.8; 73) | 77.1 (74.4; 79.8) | 70.6 (67.8; 73.5) |
| *Other* |  |  |  |
| Daily user | 1.8 (1.1; 2.5) | 1.3 (0.8; 1.9) | 1.8 (1.1; 2.5) |
| Occasional user | 1.8 (1; 2.6) | 1.3 (0.7; 1.9) | 1.8 (1; 2.5) |
| Former user | 8.4 (4.3; 12.5) | 5.9 (2.9; 8.9) | 8.3 (4.4; 12.3) |
| Never user | 87.9 (82.4; 93.5) | 91.4 (87.2; 95.6) | 88.1 (82.8; 93.5) |
| **Participation in social activities** | | | |
| *Active participation* | | | |
| Daily user | 2.6 (2.3; 3) | 2.1 (1.8; 2.4) | 2.6 (2.2; 2.9) |
| Occasional user | 2.7 (2.4; 3.1) | 2.1 (1.8; 2.4) | 2.7 (2.3; 3) |
| Former user | 14.1 (12.9; 15.4) | 10.4 (9.3; 11.4) | 13.9 (12.9; 15) |
| Never user | 80.5 (79; 82.1) | 85.4 (84.1; 86.8) | 80.8 (79.5; 82.1) |
| *Occasional* |  |  |  |
| Daily user | 2.6 (2.3; 2.9) | 2 (1.7; 2.3) | 2.5 (2.2; 2.8) |
| Occasional user | 2.7 (2.3; 3) | 2.1 (1.8; 2.3) | 2.6 (2.3; 2.9) |
| Former user | 13.6 (12.3; 14.9) | 10 (8.9; 11.1) | 13.4 (12.3; 14.5) |
| Never user | 81.2 (79.6; 82.8) | 86 (84.6; 87.4) | 81.5 (80.2; 82.9) |
| *No participation* |  |  |  |
| Daily user | 2.6 (2.3; 2.9) | 2.1 (1.8; 2.3) | 2.5 (2.2; 2.8) |
| Occasional user | 2.7 (2.4; 3) | 2.1 (1.8; 2.4) | 2.6 (2.3; 3) |
| Former user | 14. (12.9; 15) | 10.3 (9.3; 11.2) | 13.8 (13; 14.6) |
| Never user | 80.7 (79.4; 82) | 85.6 (84.3; 86.8) | 81 (80.1; 82) |

# **Table S6**. Model-adjusted prevalences of e-cigarettes with nicotine by sociodemographic characteristics, 2018-2020

|  | **2018** | **2020** |
| --- | --- | --- |
| **Age** |  |  |
| *20-39 years old* |  |  |
| Daily user | 0.9 (0.7; 1.1) | 0.9 (0.7; 1.1) |
| Occasional user | 1.3 (1.1; 1.6) | 1.3 (1.1; 1.6) |
| Former user | 20.3 (18.6; 22) | 20 (18.7; 21.4) |
| Never user | 77.5 (75.6; 79.3) | 77.7 (76.3; 79.2) |
| *40-59 years old* |  |  |
| Daily user | 0.6 (0.5; 0.8) | 0.6 (0.5; 0.8) |
| Occasional user | 0.9 (0.7; 1.1) | 0.9 (0.7; 1.1) |
| Former user | 12.8 (11.6; 13.9) | 12.6 (11.6; 13.6) |
| Never user | 85.7 (84.4; 86.9) | 85.8 (84.8; 86.9) |
| *60 to 74 years old* |  |  |
| Daily user | 0.5 (0.4; 0.7) | 0.5 (0.4; 0.6) |
| Occasional user | 0.8 (0.6; 0.9) | 0.8 (0.6; 0.9) |
| Former user | 10.1 (9.3; 11) | 10 (9.3; 10.8) |
| Never user | 88.6 (87.6; 89.5) | 88.7 (87.9; 89.5) |
| **Educational level** |  |  |
| *Lower tertile* |  |  |
| Daily user | 0.9 (0.6; 1.1) | 0.8 (0.6; 1) |
| Occasional user | 1.3 (1; 1.6) | 1.3 (1; 1.6) |
| Former user | 19.5 (17.3; 21.7) | 19 (17; 21) |
| Never user | 78.3 (75.9; 80.7) | 78.9 (76.7; 81) |
| *Middle tertile* |  |  |
| Daily user | 0.8 (0.6; 1) | 0.8 (0.6; 0.9) |
| Occasional user | 1.2 (0.9; 1.4) | 1.2 (0.9; 1.4) |
| Former user | 17.1 (15.7; 18.4) | 16.6 (15.5; 17.7) |
| Never user | 81 (79.5; 82.4) | 81.5 (80.3; 82.6) |
| *Highest tertile* |  |  |
| Daily user | 0.6 (0.4; 0.7) | 0.6 (0.4; 0.7) |
| Occasional user | 0.9 (0.7; 1.1) | 0.9 (0.7; 1) |
| Former user | 11.7 (10.6; 12.8) | 11.4 (10.5; 12.2) |
| Never user | 86.8 (85.6; 88) | 87.2 (86.3; 88.2) |
| **Marital status** |  |  |
| *Married or cohabiting* |  |  |
| Daily user | 0.7 (0.5; 0.8) | 0.7 (0.5; 0.8) |
| Occasional user | 1 (0.8; 1.2) | 1 (0.8; 1.2) |
| Former user | 13.9 (12.8; 14.9) | 13.7 (12.8; 14.5) |
| Never user | 84.4 (83.3; 85.6) | 84.7 (83.8; 85.6) |
| *Separated, widowed or divorced* | | |
| Daily user | 0.7 (0.6; 0.9) | 0.7 (0.6; 0.9) |
| Occasional user | 1.1 (0.9; 1.4) | 1.1 (0.9; 1.3) |
| Former user | 16.1 (14.7; 17.5) | 15.8 (14.7; 17) |
| Never user | 82 (80.5; 83.6) | 82.3 (81.1; 83.6) |
| **Mother tongue** |  |  |
| *Finnish* |  |  |
| Daily user | 0.7 (0.5; 0.9) | 0.7 (0.5; 0.8) |
| Occasional user | 1 (0.8; 1.2) | 1 (0.8; 1.2) |
| Former user | 14.5 (13.4; 15.5) | 14.3 (13.5; 15) |
| Never user | 83.8 (82.7; 84.9) | 84 (83.2; 84.8) |
| *Swedish* |  |  |
| Daily user | 0.8 (0.6; 1.1) | 0.8 (0.6; 1) |
| Occasional user | 1.2 (1; 1.5) | 1.2 (1; 1.5) |
| Former user | 18.5 (15.7; 21.3) | 18.3 (15.6; 21.1) |
| Never user | 79.4 (76.4; 82.5) | 79.6 (76.7; 82.6) |
| *Other* |  |  |
| Daily user | 0.8 (0.5; 1) | 0.8 (0.5; 1) |
| Occasional user | 1.1 (0.7; 1.5) | 1.1 (0.7; 1.5) |
| Former user | 16.5 (10.2; 22.9) | 16.3 (10.2; 22.5) |
| Never user | 81.6 (74.7; 88.5) | 81.8 (75.1; 88.5) |
| **Participation in social activities** | | |
| *Active participation* |  |  |
| Daily user | 0.6 (0.5; 0.8) | 0.6 (0.5; 0.7) |
| Occasional user | 0.9 (0.7; 1.1) | 0.9 (0.7; 1) |
| Former user | 12.2 (10.9; 13.5) | 11.9 (10.7; 13) |
| Never user | 86.3 (84.9; 87.7) | 86.7 (85.4; 87.9) |
| *Occasional* |  |  |
| Daily user | 0.7 (0.5; 0.8) | 0.6 (0.5; 0.8) |
| Occasional user | 1 (0.7; 1.2) | 0.9 (0.7; 1.1) |
| Former user | 13.4 (12; 14.8) | 13.1 (11.8; 14.3) |
| Never user | 85 (83.5; 86.5) | 85.4 (84; 86.7) |
| *No participation* |  |  |
| Daily user | 0.8 (0.6; 1) | 0.8 (0.6; 0.9) |
| Occasional user | 1.1 (0.9; 1.4) | 1.1 (0.9; 1.3) |
| Former user | 16.6 (15.3; 17.8) | 16.2 (15.2; 17.2) |
| Never user | 81.5 (80.2; 82.9) | 81.9 (80.9; 83) |

# **Table S7**. Model-adjusted prevalences of e-cigarettes without nicotine by sociodemographic characteristics, 2018-2020

|  | **2018** | **2020** |
| --- | --- | --- |
| **Age** |  |  |
| *20-39 years old* |  |  |
| Daily user | 0.1 (0.1; 0.2) | 0.1 (0.1; 0.2) |
| Occasional user | 0.6 (0.4; 0.8) | 0.6 (0.4; 0.8) |
| Former user | 18.3 (16.6; 20) | 18.1 (16.7; 19.4) |
| Never user | 80.9 (79.2; 82.7) | 81.2 (79.8; 82.6) |
| *40-59 years old* |  |  |
| Daily user | 0.1 (0; 0.1) | 0.1 (0; 0.1) |
| Occasional user | 0.4 (0.3; 0.6) | 0.4 (0.3; 0.6) |
| Former user | 11.9 (10.7; 13) | 11.7 (10.7; 12.7) |
| Never user | 87.6 (86.4; 88.8) | 87.8 (86.8; 88.8) |
| *60 years and older* |  |  |
| Daily user | 0.1 (0; 0.1) | 0.1 (0; 0.1) |
| Occasional user | 0.4 (0.3; 0.5) | 0.4 (0.3; 0.5) |
| Former user | 9.9 (9; 10.8) | 9.7 (9; 10.5) |
| Never user | 89.7 (88.8; 90.5) | 89.8 (89; 90.6) |
| **Educational level** |  |  |
| *Lower tertile* |  |  |
| Daily user | 0.1 (0.1; 0.2) | 0.1 (0.1; 0.2) |
| Occasional user | 0.6 (0.4; 0.8) | 0.6 (0.4; 0.8) |
| Former user | 17.6 (15.4; 19.7) | 17.1 (15.1; 19) |
| Never user | 81.7 (79.5; 84) | 82.2 (80.2; 84.2) |
| *Middle tertile* |  |  |
| Daily user | 0.1 (0.1; 0.2) | 0.1 (0.1; 0.1) |
| Occasional user | 0.5 (0.4; 0.7) | 0.5 (0.4; 0.7) |
| Former user | 15.8 (14.4; 17.2) | 15.4 (14.3; 16.4) |
| Never user | 83.6 (82.1; 85) | 84 (82.9; 85.1) |
| *Highest tertile* |  |  |
| Daily user | 0.1 (0; 0.1) | 0.1 (0; 0.1) |
| Occasional user | 0.4 (0.3; 0.5) | 0.4 (0.3; 0.5) |
| Former user | 10.9 (9.8; 12) | 10.6 (9.7; 11.4) |
| Never user | 88.6 (87.5; 89.8) | 89 (88.1; 89.9) |
| **Marital status** |  |  |
| *Married or cohabiting* | | |
| Daily user | 0.1 (0; 0.1) | 0.1 (0; 0.1) |
| Occasional user | 0.5 (0.3; 0.6) | 0.5 (0.3; 0.6) |
| Former user | 12.9 (11.8; 13.9) | 12.6 (11.8; 13.4) |
| Never user | 86.6 (85.5; 87.7) | 86.8 (86; 87.7) |
| *Separated, widowed or divorced* | | |
| Daily user | 0.1 (0.1; 0.1) | 0.1 (0.1; 0.1) |
| Occasional user | 0.5 (0.4; 0.7) | 0.5 (0.4; 0.7) |
| Former user | 14.9 (13.5; 16.4) | 14.7 (13.5; 15.8) |
| Never user | 84.4 (83; 85.9) | 84.7 (83.5; 85.9) |
| **Mother tongue** |  |  |
| *Finnish* |  |  |
| Daily user | 0.1 (0.1; 0.1) | 0.1 (0.1; 0.1) |
| Occasional user | 0.5 (0.3; 0.6) | 0.5 (0.3; 0.6) |
| Former user | 13.4 (12.4; 14.4) | 13.2 (12.5; 13.9) |
| Never user | 86 (85; 87.1) | 86.2 (85.5; 87) |
| *Swedish* |  |  |
| Daily user | 0.1 (0.1; 0.2) | 0.1 (0.1; 0.2) |
| Occasional user | 0.6 (0.4; 0.8) | 0.6 (0.4; 0.8) |
| Former user | 17 (14.; 20.1) | 16.8 (13.9; 19.7) |
| Never user | 82.3 (79.1; 85.4) | 82.5 (79.5; 85.5) |
| *Other* |  |  |
| Daily user | 0.1 (0.1; 0.2) | 0.1 (0.1; 0.2) |
| Occasional user | 0.5 (0.3; 0.8) | 0.5 (0.3; 0.8) |
| Former user | 16 (9.4; 22.6) | 15.8 (9.4; 22.2) |
| Never user | 83.3 (76.5; 90.2) | 83.6 (77; 90.2) |
| **Participation in social activities** | | |
| *Active participation* |  |  |
| Daily user | 0.1 (0; 0.1) | 0.1 (0; 0.1) |
| Occasional user | 0.4 (0.3; 0.5) | 0.4 (0.3; 0.5) |
| Former user | 11.2 (9.9; 12.5) | 10.9 (9.8; 12) |
| Never user | 88.3 (87; 89.6) | 88.6 (87.5; 89.8) |
| *Occasional* |  |  |
| Daily user | 0.1 (0; 0.1) | 0.1 (0; 0.1) |
| Occasional user | 0.5 (0.3; 0.6) | 0.4 (0.3; 0.6) |
| Former user | 12.7 (11.3; 14.2) | 12.4 (11.1; 13.6) |
| Never user | 86.7 (85.2; 88.2) | 87.1 (85.8; 88.4) |
| *No participation* |  |  |
| Daily user | 0.1 (0.1; 0.1) | 0.1 (0.1; 0.1) |
| Occasional user | 0.5 (0.4; 0.7) | 0.5 (0.4; 0.7) |
| Former user | 15.3 (14.1; 16.6) | 14.9 (14.; 15.9) |
| Never user | 84 (82.7; 85.3) | 84.5 (83.5; 85.4) |

# **Table S8**. Model-adjusted prevalences of NRT products by sociodemographic characteristics, 2018-2020

|  | **2018** | **2020** |
| --- | --- | --- |
| **Age** |  |  |
| *20-39 years old* |  |  |
| Daily user | 1.6 (1.4; 1.9) | 1.7 (1.5; 1.9) |
| Occasional user | 3.5 (3.1; 3.9) | 3.8 (3.4; 4.1) |
| Former user | 17.5 (15.4; 19.5) | 19.1 (17.7; 20.5) |
| Never user | 77.4 (75; 79.8) | 75.4 (73.8; 77.1) |
| *40-59 years old* |  |  |
| Daily user | 1.6 (1.4; 1.8) | 1.7 (1.5; 1.9) |
| Occasional user | 3.4 (3; 3.8) | 3.6 (3.2; 4) |
| Former user | 16.9 (15.3; 18.5) | 17.9 (16.8; 19.1) |
| Never user | 78.1 (76.2; 80) | 76.8 (75.5; 78.1) |
| *60 to 74 years old* |  |  |
| Daily user | 1.4 (1.2; 1.5) | 1.2 (1; 1.3) |
| Occasional user | 2.9 (2.6; 3.2) | 2.5 (2.3; 2.8) |
| Former user | 13.6 (12.4; 14.8) | 11.3 (10.6; 12.1) |
| Never user | 82.1 (80.7; 83.5) | 85 (84; 85.9) |
| **Educational level** |  |  |
| *Lower tertile* |  |  |
| Daily user | 1.8 (1.5; 2) | 1.8 (1.5; 2) |
| Occasional user | 3.9 (3.5; 4.4) | 3.9 (3.5; 4.3) |
| Former user | 20 (17.9; 22.1) | 20.1 (18.2; 22) |
| Never user | 74.3 (71.9; 76.7) | 74.2 (72; 76.4) |
| *Middle tertile* |  |  |
| Daily user | 1.7 (1.5; 1.9) | 1.7 (1.5; 1.9) |
| Occasional user | 3.7 (3.3; 4.1) | 3.7 (3.3; 4.1) |
| Former user | 18.5 (17.1; 19.9) | 18.6 (17.5; 19.7) |
| Never user | 76.1 (74.5; 77.7) | 76 (74.7; 77.2) |
| *Highest tertile* |  |  |
| Daily user | 1.4 (1.2; 1.5) | 1.3 (1.2; 1.5) |
| Occasional user | 2.9 (2.6; 3.2) | 2.9 (2.6; 3.2) |
| Former user | 13.3 (12.3; 14.4) | 13.5 (12.6; 14.3) |
| Never user | 82.4 (81.1; 83.7) | 82.3 (81.2; 83.3) |
| **Marital status** |  |  |
| *Married or cohabiting* |  |  |
| Daily user | 1.5 (1.3; 1.7) | 1.5 (1.3; 1.7) |
| Occasional user | 3.3 (2.9; 3.6) | 3.3 (3; 3.6) |
| Former user | 15.8 (14.7; 16.8) | 16 (15.2; 16.9) |
| Never user | 79.4 (78.2; 80.7) | 79.1 (78.2; 80.1) |
| *Separated, widowed or divorced* | | |
| Daily user | 1.6 (1.4; 1.8) | 1.6 (1.4; 1.8) |
| Occasional user | 3.4 (3.1; 3.8) | 3.4 (3.1; 3.8) |
| Former user | 16.7 (15.4; 18.1) | 17 (15.9; 18.1) |
| Never user | 78.3 (76.7; 79.9) | 78 (76.6; 79.3) |
| **Mother tongue** |  |  |
| *Finnish* |  |  |
| Daily user | 1.5 (1.3; 1.7) | 1.5 (1.4; 1.7) |
| Occasional user | 3.3 (3; 3.6) | 3.3 (3; 3.6) |
| Former user | 16 (15; 17) | 16.3 (15.6; 17.1) |
| Never user | 79.2 (78; 80.3) | 78.8 (78; 79.6) |
| *Swedish* |  |  |
| Daily user | 1.7 (1.4; 1.9) | 1.7 (1.4; 1.9) |
| Occasional user | 3.7 (3.1; 4.2) | 3.7 (3.2; 4.2) |
| Former user | 18.4 (15.5; 21.4) | 18.8 (15.9; 21.6) |
| Never user | 76.2 (72.7; 79.7) | 75.9 (72.5; 79.2) |
| *Other* |  |  |
| Daily user | 1.6 (1.2; 2.1) | 1.6 (1.2; 2.1) |
| Occasional user | 3.5 (2.4; 4.5) | 3.5 (2.5; 4.5) |
| Former user | 17.3 (10.3; 24.3) | 17.6 (10.8; 24.5) |
| Never user | 77.6 (69.2; 86) | 77.2 (69; 85.5) |
| **Participation in social activities** | | |
| *Active participation* |  |  |
| Daily user | 1.3 (1.2; 1.5) | 1.3 (1.2; 1.5) |
| Occasional user | 2.8 (2.5; 3.1) | 2.8 (2.5; 3.1) |
| Former user | 13.1 (11.9; 14.4) | 13.3 (12.2; 14.4) |
| Never user | 82.7 (81.2; 84.2) | 82.5 (81.2; 83.9) |
| *Occasional* |  |  |
| Daily user | 1.5 (1.3; 1.7) | 1.5 (1.3; 1.7) |
| Occasional user | 3.2 (2.8; 3.5) | 3.2 (2.8; 3.5) |
| Former user | 15.4 (13.9; 16.8) | 15.5 (14.3; 16.7) |
| Never user | 80 (78.3; 81.7) | 79.8 (78.3; 81.3) |
| *No participation* |  |  |
| Daily user | 1.7 (1.5; 1.9) | 1.7 (1.5; 1.9) |
| Occasional user | 3.6 (3.2; 4) | 3.6 (3.2; 3.9) |
| Former user | 18.1 (16.9; 19.4) | 18.3 (17.3; 19.2) |
| Never user | 76.6 (75.2; 78) | 76.5 (75.4; 77.6) |

# **Table S9**. Model-adjusted prevalences of any daily tobacco or nicotine use by sociodemographic characteristics, 2018-2020

|  | **2018** | **2020** |
| --- | --- | --- |
| **Age** |  |  |
| *20-39 years old* |  |  |
| Any daily tobacco or nicotine use | 17.3 (15.7; 18.9) | 16.2 (14.9; 17.5) |
| None reported | 82.7 (81.1; 84.3) | 83.8 (82.5; 85.1) |
| *40-59 years old* |  |  |
| Any daily tobacco or nicotine use | 16.6 (15.2; 18) | 15.6 (14.5; 16.6) |
| None reported | 83.4 (82; 84.8) | 84.4 (83.4; 85.5) |
| *60 to 74 years old* |  |  |
| Any daily tobacco or nicotine use | 12 (11; 13) | 11.2 (10.4; 12.1) |
| None reported | 88 (87; 89) | 88.8 (87.9; 89.6) |
| **Educational level** |  |  |
| *Lower tertile* |  |  |
| Any daily tobacco or nicotine use | 24.3 (21.7; 26.9) | 22 (19.8; 24.2) |
| None reported | 75.7 (73.1; 78.3) | 78 (75.8; 80.2) |
| *Middle tertile* |  |  |
| Any daily tobacco or nicotine use | 20.3 (18.7; 21.9) | 18.3 (17.2; 19.5) |
| None reported | 79.7 (78.1; 81.3) | 81.7 (80.5; 82.8) |
| *Highest tertile* |  |  |
| Any daily tobacco or nicotine use | 10 (9; 11) | 8.9 (8.1; 9.7) |
| None reported | 90 (89; 91) | 91.1 (90.3; 91.9) |
| **Marital status** |  |  |
| *Married or cohabiting* |  |  |
| Any daily tobacco or nicotine use | 13.6 (12.6; 14.6) | 12.5 (11.8; 13.3) |
| None reported | 86.4 (85.4; 87.4) | 87.5 (86.7; 88.2) |
| *Separated, widowed or divorced* |  |  |
| Any daily tobacco or nicotine use | 19.6 (17.9; 21.2) | 18.2 (16.9; 19.4) |
| None reported | 80.4 (78.8; 82.1) | 81.8 (80.6; 83.1) |
| **Mother tongue** |  |  |
| *Finnish* |  |  |
| Any daily tobacco or nicotine use | 15.5 (14.5; 16.6) | 14.5 (13.8; 15.3) |
| None reported | 84.5 (83.4; 85.5) | 85.5 (84.7; 86.2) |
| *Swedish* |  |  |
| Any daily tobacco or nicotine use | 18.2 (14.4; 22.1) | 17.1 (13.7; 20.6) |
| None reported | 81.8 (77.9; 85.6) | 82.9 (79.4; 86.3) |
| *Other* |  |  |
| Any daily tobacco or nicotine use | 10 (6; 14.) | 9.3 (5.6; 13) |
| None reported | 90 (86; 94) | 90.7 (87; 94.4) |
| **Participation in social activities** |  |  |
| *Active participation* |  |  |
| Any daily tobacco or nicotine use | 9.7 (8.5; 10.9) | 8.8 (7.8; 9.8) |
| None reported | 90.3 (89.1; 91.5) | 91.2 (90.2; 92.2) |
| *Occasional* |  |  |
| Any daily tobacco or nicotine use | 12.3 (10.9; 13.6) | 11.2 (10; 12.4) |
| None reported | 87.7 (86.4; 89.1) | 88.8 (87.6; 90) |
| *No participation* |  |  |
| Any daily tobacco or nicotine use | 20.1 (18.7; 21.5) | 18.5 (17.5; 19.5) |
| None reported | 79.9 (78.5; 81.3) | 81.5 (80.5; 82.5) |
