# Supplementary material for: Changes in prevalence and sociodemographic correlates of tobacco and nicotine use in Finland during the COVID-19 pandemic
Source: Eur J Public Health. 2023 Jul 3;33(5):844–50. doi: 10.1093/eurpub/ckad104 (PMC10567130; doi:10.1093/eurpub/ckad104)
Supplement: ckad104_Supplementary_Data [file ckad104_supplementary_data.zip › ckad104_Supplementary_Data/ejph-2023-02-om-0064-File004.pdf]

Changes in tobacco and nicotine use during the COVID-19 pandemic: Statistical code part 2

Sebastián Peña et al

July 7 2022

This R file provides the code to carry out the harmonization of variables and the plots. The main analyses were carried out in Stata version 17 (see .do file below).

Loading packages and dataset

```
library(readxl)
library(foreign)
library(haven)
library(googlesheets4)
library(styler)
library(tidyverse)
library(knitr)
library(kableExtra)
library(tidyr)
library(janitor)
library(gtools)
library(ggridges)
library(gridExtra)
library(ggfortify)
library(tableone)
library(pollster)
library(naniar)
library(survey)
library(survival)
library(biostat3)
library(Epi)
library(marginaleffects)
library(margins)
library(ggsci)
library(extrafont)
library(stringr)
library(cowplot)
library(scales)
library(RStata)


#font_import()
```

```
#loadfonts(device = "win")

options(scipen=999)

# Loading FinSote dataset
finsote2018 <- read_sas("//helfs01.thl.fi/groups4/Tobrisk_FinSote/10147/FinSote2018/data_f181.sas7bdat"
finsote2019 <- read_sas("//helfs01.thl.fi/groups4/Tobrisk_FinSote/10147/FinSote2019/data_f191.sas7bdat"
finsote2020 <- read_sas("//helfs01.thl.fi/groups4/Tobrisk_FinSote/10147/FinSote2020/data_f201.sas7bdat"
finsote2020_dates <- read_excel("//helfs01.thl.fi/groups4/Tobrisk_FinSote/10147/FinSote2020/F201_respons
finsote2020covid <- read_sas("//helfs01.thl.fi/groups4/Tobrisk_FinSote/10147/FinSote2020_covid/data_f20
```

ggplot theme functions

These functions are required for publication quality plots

```
theme_Publication <- function(base_size=12, base_family="Arial") {
      library(grid)
      library(ggthemes)
      (theme_foundation(base_size=base_size, base_family=base_family)
       + theme(plot.title = element_text(face = "bold",
                                         size = rel(1.2), hjust = 0.5),
               text = element_text(),
               panel.background = element_rect(colour = NA),
               plot.background = element_rect(colour = NA),
               panel.border = element_rect(colour = NA),
               axis.title = element_text(face = "bold",size = rel(1)),
               axis.title.y = element_text(angle=90,vjust =2),
               axis.title.x = element_text(vjust = -0.2),
               axis.text = element_text(size=10),
               axis.line = element_line(colour="black"),
               axis.ticks = element_line(),
               panel.grid.major = element_blank(),
               panel.grid.minor = element_blank(),
               legend.key = element_rect(colour = NA),
               legend.position = "bottom",
               legend.direction = "horizontal",
               legend.key.size= unit(0.2, "cm"),
               legend.margin = unit(0, "cm"),
               legend.title = element_text(face="italic"),
               plot.margin=unit(c(10,5,5,5),"mm"),
               strip.background=element_rect(colour="#f0f0f0",fill="#f0f0f0"),
               strip.text = element_text(face="bold")
          ))

}

scale_fill_Publication <- function(...){
      library(scales)
      discrete_scale("fill","Publication",manual_pal(values =
c("#386cb0","#fdb462","#7fc97f","#ef3b2c","#662506","#a6cee3","#fb9a99","#984ea3",
  "#ffff33")), ...)
```

```
}

scale_colour_Publication <- function(...){
      library(scales)
      discrete_scale("colour","Publication",manual_pal(values =
c("#386cb0","#fdb462","#7fc97f","#ef3b2c","#662506","#a6cee3","#fb9a99","#984ea3",
  "#ffff33")), ...)

}
```

Management of variables

We harmonised all three datasets according to the variables described in the study protocol. FinSote 2018 and 2020 have identical questions for the variables of interest; we, therefore, created a unique dataset appending both datasets. FinSote 2019 was conducted in conjunction with EHIS 3 and questions are not always identical but can produce harmonized variables. This is particularly true for smoking questions. The code will note when questions are identical or different, more information can be found in the Supplementary Appendix.

The section creates a pooled dataset (which includes participants with missing data) of 49186 participants, including all participants in 2018, 2019 and 2020 who provided consent for data linkage.

```
# FINSOTE 2018 and 2020
# Creating data id FinSote 2018 and subset with key variables
finsote2018_brief <- finsote2018 %>%
  dplyr::select(ID, GUMM85ID, consent_reg, maritalstatus, educ_years, IKA2,
                height_cm, weight_kg, involvement_attend_j, smoke_cur_never,
                smoke_altprod_snus, smoke_altprod_nic_ecig,
                smoke_altprod_non_nic_ecig, smoke_altprod_nic_substitute,
                smoke_altprod_recipe, smoke_altprod_other, Sukupuoli,
                kieliryhma, fs_shp_koodi, fs_shp_nimi_fi, rg_N1, w_analysis1,
                rg_stratum1, w_expansion1, rg_stratum) %>%
  rename(rg_N_suomi=rg_N1) %>%
  rename(w_analysis_suomi=w_analysis1) %>%
  rename(rg_stratum_suomi=rg_stratum1) %>%
  rename(w_expansion_suomi=w_expansion1) %>%
  mutate(dataid=2018) %>%
  mutate(age_cont=IKA2) %>%
  mutate(educ_tertiles = ntile(educ_years,3)) ##this creates specific education
##tertiles for each year


# Appending datasets with different data types creates problems
finsote2018_brief$Sukupuoli <- as.factor(finsote2018_brief$Sukupuoli)

# Creating data id FinSote 2020 and subset with key variables
finsote2020_brief <- finsote2020 %>%
  dplyr::select(ID, GUMM85ID, maritalstatus, educ_years, IKA2, height_cm, weight_kg,
                involvement_attend_j, smoke_cur_never, smoke_altprod_snus,
                smoke_altprod_nic_ecig, smoke_altprod_non_nic_ecig,
                smoke_altprod_nic_substitute,
                sukupuoli, kieliryhma, fs_shp_koodi, rg_N_suomi, w_analysis_suomi,
```

```
                rg_stratum_suomi, w_expansion_suomi, rg_stratum_hyvinvointialue) %>%
  rename(Sukupuoli=sukupuoli) %>%
  mutate(dataid=2020) %>%
  mutate(age_cont=IKA2) %>%
  rename(rg_stratum=rg_stratum_hyvinvointialue) %>%
  mutate(educ_tertiles = ntile(educ_years,3)) ##this creates specific education
##tertiles for each year

# Appending datasets with different data types creates problems
finsote2020_brief$Sukupuoli <- as.factor(finsote2020_brief$Sukupuoli)

# Appending both datasets
finsote1820 <- bind_rows(finsote2018_brief, finsote2020_brief)  #dplyr works better than smartbind
miss_var_summary(finsote1820)                                   #now looks good

finsote1820 <- finsote1820 %>%
  mutate(mother_tongue = kieliryhma) %>%
  mutate(sex=Sukupuoli)

# Checking class and levels of key variables
class(finsote1820$smoke_cur_never)  #numeric
class(finsote1820$maritalstatus)    #numeric
class(finsote1820$educ_years)       #numeric. ready for use
class(finsote1820$sex)              #factor
class(finsote1820$mother_tongue)    #numeric

# Tobacco use. Convert to factor, creating levels
## Smoking
finsote1820$smoking_status <- as.factor(finsote1820$smoke_cur_never)
finsote1820$smoking_status <-  recode_factor(finsote1820$smoking_status,
                                             `1`="daily smoker",
                                             `2`="occasional", `3`="former smoker",
                                             `4`="never smoker")

## Snus
finsote1820$snus_status <- as.factor(finsote1820$smoke_altprod_snus)
finsote1820$snus_status <-  recode_factor(finsote1820$snus_status,
                                          `1`="daily user",
                                          `2`="occasional", `3`="former user",
                                          `4`="never user")

## E-cig with nicotine
finsote1820$ecig_nic_status <- as.factor(finsote1820$smoke_altprod_nic_ecig)
finsote1820$ecig_nic_status <-  recode_factor(finsote1820$ecig_nic_status,
                                          `1`="daily user",
                                          `2`="occasional", `3`="former user",
                                          `4`="never user")

## E-cig without nicotine
finsote1820$ecig_nonic_status <- as.factor(finsote1820$smoke_altprod_non_nic_ecig)
finsote1820$ecig_nonic_status <-  recode_factor(finsote1820$ecig_nonic_status,
                                          `1`="daily user",
                                          `2`="occasional", `3`="former user",
```

```r
                                          `4`="never user")

## Nicotine replacement therapy
finsote1820$nrt_status <- as.factor(finsote1820$smoke_altprod_nic_substitute)
finsote1820$nrt_status <-  recode_factor(finsote1820$nrt_status,
                                         `1`="daily user",
                                         `2`="occasional", `3`="former user",
                                         `4`="never user")

# Sociodemographic characteristics

## Marital status. Convert to factor, create categorical levels
finsote1820$maritalstatus_bin <- as.factor(finsote1820$maritalstatus)
finsote1820$maritalstatus_bin <-  recode_factor(finsote1820$maritalstatus_bin,
                                                `1`="married or cohabiting",
                                                `2`="married or cohabiting",
                                                `3`="separated, single or widowed",
                                                `4`="separated, single or widowed",
                                                `5`="separated, single or widowed")

## Mother tongue
finsote1820$mother_tongue <- as.factor(finsote1820$mother_tongue)
finsote1820$mother_tongue <-  recode_factor(finsote1820$mother_tongue, `1`="finnish",
                                            `2`="swedish", `3`="other", `4`="other")

## Sex. Needs no modification. 1==male and 2==female
##Years of education. Needs no modification, continuous variable.

## Social participation
finsote1820$involvement_attend_j <- as.factor(finsote1820$involvement_attend_j)
finsote1820$involvement_attend_j <-  recode_factor(finsote1820$involvement_attend_j,
                                                   `1`="no participation", `2`="active",
                                                   `3`="occasional")

## BMI
class(finsote1820$height_cm) #with the merge, both variables convert to character
class(finsote1820$weight_kg) #with the merge, both variables convert to character
finsote1820$height_cm <- as.numeric(finsote1820$height_cm)
finsote1820$weight_kg <- as.numeric(finsote1820$weight_kg)
finsote1820 <- finsote1820 %>%
  mutate(bmi = weight_kg/(height_cm*height_cm/10000))

# Convert complex survey variables
finsote1820$rg_N_suomi <- as.numeric(finsote1820$rg_N_suomi)
finsote1820$rg_stratum_suomi <- as.numeric(finsote1820$rg_stratum_suomi)
finsote1820$w_analysis_suomi <- as.numeric(finsote1820$w_analysis_suomi)
finsote1820$w_expansion_suomi <- as.numeric(finsote1820$w_expansion_suomi)
miss_var_summary(finsote2020_brief)

# Check variable types
class(finsote1820$maritalstatus_bin) #factor
class(finsote1820$sex) #factor
class(finsote1820$rg_N_suomi) #numeric
```

```
# Dataset combining FinSote 2018 and 2020 ready

# FINSOTE 2019 (restricted to participants aged 20+ for comparability)
## Creating data id FinSote 2019 and subset with key variables
finsote2019_brief <- finsote2019 %>%
  dplyr::select(ID, GUMM85ID, maritalstatus, educ_years, IKA2, height_cm, weight_kg,
                involvement_attend_j, ehis_smoking_current, ehis_smoking_product,
                ehis_smoking_cigarettes,    smoke_oneyear, smoke_years,
                ehis_smoke_indoor,smoke_snus_now2,  smoke_ecig_now, Sukupuoli,
                kieliryhma, fs_shp_koodi,fs_shp_nimi_fi, rg_N, rg_stratum,
                w_analysis, w_expansion) %>%
  rename(rg_N_suomi=rg_N) %>%
  rename(w_analysis_suomi=w_analysis) %>%
  mutate(rg_stratum_suomi=rg_stratum) %>%
  rename(w_expansion_suomi=w_expansion) %>%
  mutate(age_cont=IKA2) %>%
  mutate(dataid=2019) %>%
  filter(age_cont>=20) %>% ## excludes participants younger than 20 for comparability
  mutate(educ_tertiles = ntile(educ_years,3)) ##this creates specific education
##tertiles for each year

finsote2019_brief <- finsote2019_brief %>%
  mutate(mother_tongue = kieliryhma) %>%
  mutate(sex=Sukupuoli)

# Checking class and levels of key variables
class(finsote2019_brief$maritalstatus)    #numeric
class(finsote2019_brief$educ_years)       #numeric. ready for use
class(finsote2019_brief$sex)              #character
class(finsote2019_brief$mother_tongue)    #numeric

## All variables are identical to FinSote 2018 and 2020 except for smoking
class(finsote2019_brief$ehis_smoking_current)
class(finsote2019_brief$ehis_smoking_product)
class(finsote2019_brief$smoke_oneyear)

class(finsote2019_brief$smoke_snus_now2)
class(finsote2019_brief$smoke_ecig_now)
table(finsote2019_brief$ehis_smoking_current, useNA = "ifany")
table(finsote2019_brief$ehis_smoking_current, finsote2019_brief$smoke_oneyear,
      useNA = "ifany")
table(finsote2019_brief$smoke_oneyear, useNA = "ifany")

## Smoking (see Supplementary Appendix for more details)
### With question 84, we can break down the "not at all" group into never and former smokers
finsote2019_brief <- finsote2019_brief %>%
  mutate(smoking_status=ehis_smoking_current) %>%
  mutate(smoking_status=replace(smoking_status, smoking_status==3 & smoke_oneyear==1,
                                5))   %>% #replaces with "former smoker"
  mutate(smoking_status=replace(smoking_status, smoking_status==3 & smoke_oneyear==2,
                                4))  #replaces with "never smoker" %>%

finsote2019_brief <- finsote2019_brief %>%
```

```
  mutate(smoking_status=replace(smoking_status, smoking_status==3, NA))  #replaces
##with NA those who answered 3 in question 81 but not answered 84

### Quality checks
table(finsote2019_brief$ehis_smoking_current, finsote2019_brief$smoke_oneyear)
table(finsote2019_brief$smoking_status, finsote2019_brief$smoke_oneyear,
      useNA = "ifany") #looks good
table(finsote2019_brief$smoking_status)

### Converting into factor and labeling
finsote2019_brief$smoking_status <- as.factor(finsote2019_brief$smoking_status)
finsote2019_brief$smoking_status <-  recode_factor(finsote2019_brief$smoking_status,
                                         `1`="daily smoker",
                                         `2`="occasional",
                                         `4`="former smoker",
                                         `5`="never smoker")

### Quality checks
table(finsote2019_brief$smoking_status, finsote2019_brief$ehis_smoking_current,
      useNA = "ifany")
table(finsote2019_brief$smoking_status, finsote2019_brief$smoke_oneyear,
      useNA = "ifany") #looks good
table(finsote2019_brief$smoking_status)

### Snus and e-cigarettes
finsote2019_brief$snus_status <- as.factor(finsote2019_brief$smoke_snus_now2)
finsote2019_brief$snus_status <-  recode_factor(finsote2019_brief$snus_status,
                                       `1`="daily user",
                                       `2`="occasional",
                                       `3`="former user",
                                       `4`="never user")

finsote2019_brief$ecig_status <- as.factor(finsote2019_brief$smoke_ecig_now)
finsote2019_brief$ecig_status <-  recode_factor(finsote2019_brief$ecig_status,
                                       `1`="daily smoker",
                                       `2`="occasional",
                                       `3`="former user",
                                       `4`="never user")

### Quality checks
table(finsote2019_brief$snus_status, finsote2019_brief$smoke_snus_now2,
      useNA = "ifany")
table(finsote2019_brief$ecig_status, finsote2019_brief$smoke_ecig_now,
      useNA = "ifany") #looks good

## Confounders
## Marital status. Convert to factor, create categorical levels
finsote2019_brief$maritalstatus_bin <- as.factor(finsote2019_brief$maritalstatus)
finsote2019_brief$maritalstatus_bin <-  recode_factor(finsote2019_brief$maritalstatus_bin,
                                            `1`="married or cohabiting",
                                            `2`="married or cohabiting",
                                            `3`="separated, single or widowed",
                                            `4`="separated, single or widowed",
```

```
                                           `5`="separated, single or widowed")

## Mother tongue
finsote2019_brief$mother_tongue <- as.factor(finsote2019_brief$mother_tongue)
finsote2019_brief$mother_tongue <-  recode_factor(finsote2019_brief$mother_tongue,
                                                 `1`="finnish", `2`="swedish",
                                                 `3`="other", `4`="other")

## Sex.

## Social participation
finsote2019_brief$involvement_attend_j <- as.factor(finsote2019_brief$involvement_attend_j)
finsote2019_brief$involvement_attend_j <-  recode_factor(finsote2019_brief$involvement_attend_j,
            `1`="no participation", `2`="active", `3`="occasional")

# Solving data type incompatibilities before appending
finsote1820$involvement_attend_j <- as.factor(finsote1820$involvement_attend_j)

# Creating final baseline dataset
finsote <- bind_rows(finsote1820, finsote2019_brief)
miss_var_summary(finsote) #finally looks good

## Creates new variable age as categorical
finsote <- finsote %>%
  mutate(age_cat = cut(age_cont, breaks = c(-Inf,39,59,Inf)))

### Checks
table(finsote$age_cont, finsote$age_cat)
finsote$educ_tertiles <-  recode_factor(finsote$educ_tertiles,
                                       `1`="Lowest tertile",
                                       `2`="Middle tertile",
                                       `3`="Highest tertile")

## Checks educational tertiles
finsote %>%
    tabyl(educ_years, educ_tertiles, dataid) %>%
  kable(digits = 1, caption = "Events by smoking status and survey wave",
        format = "html")
table(finsote$dataid, finsote$educ_tertiles)
```

Exploring missing data

We used several functions of the package *naniar* to explore missing data patterns.

Out of 49186 participants, 10.1% had missing data in any of the variables. The variables with highest frequency of missing data were years of education, smoking status and BMI.

```
# Creates a subset so that miss_var_summary only shows the variables of interest
finsote_missing <- finsote %>%
  dplyr::select(dataid, rg_N_suomi, w_analysis_suomi, rg_stratum_suomi,
                fs_shp_koodi,age_cont, sex,smoking_status, educ_years,
                maritalstatus_bin, mother_tongue,
```

```
                involvement_attend_j, bmi)
finsote_missing %>%
    group_by(dataid) %>%
  miss_var_summary()


prop_complete(finsote_missing)
pct_complete(finsote_missing)
pct_miss(finsote_missing)
pct_complete_case(finsote_missing)
pct_miss_case(finsote_missing)
miss_case_table(finsote_missing)
```

Exploratory data analysis

```
# Exploratory cross tabs
## Outcomes
table(finsote$smoking_status, finsote$dataid)
table(finsote$snus_status, finsote$dataid) #prevalence of snus use is (as we knew) low
table(finsote$ecig_nic_status, finsote$dataid) #very few users
table(finsote$nrt_status, finsote$dataid) #very few users

## Sociodemographic characteristics
table(finsote$age_cont, finsote$dataid)
table(finsote$sex, finsote$dataid)
table(finsote$maritalstatus_bin, finsote$dataid)
table(finsote$educ_years, finsote$dataid)
table(finsote$mother_tongue, finsote$dataid)
table(finsote$involvement_attend_j, finsote$dataid)
table(finsote$bmi, finsote$dataid)

#Ridgeplots for continuous variables
## Years of education
finsote$dataid <- as.factor(finsote$dataid) #this graphs require a factor variable
ggplot(finsote, aes(x = educ_years, y = dataid, fill = dataid)) +
  geom_density_ridges(scale = 2, rel_min_height = 0.01) +
  labs(title = 'years of education')
ggsave(file="educ_years.png", units="cm", width=25, height=15, dpi=600) #looks good

## Age
ggplot(finsote, aes(x =age_cont, y = dataid, fill = dataid)) +
  geom_density_ridges(scale = 2, rel_min_height = 0.01) +
  labs(title = 'Age')
ggsave(file="age.png", units="cm", width=25, height=15, dpi=600) #looks good
```

Incorporates survey design

```
# Creates subset of key variables
finsotecc <- finsote %>%
```

```
  dplyr::select(ID, rg_N_suomi, w_analysis_suomi, rg_stratum_suomi, rg_stratum,
                dataid, fs_shp_koodi, age_cont, age_cat, sex, smoking_status,
                snus_status, ecig_nic_status, ecig_nonic_status, nrt_status,
                educ_years, educ_tertiles, maritalstatus_bin, mother_tongue,
                involvement_attend_j, bmi) %>%
   filter(complete.cases(smoking_status))


finsote_males <- finsotecc %>%
  filter(sex==1)

finsote_females <- finsotecc %>%
  filter(sex==2)

# Combines daily and occasional users
table(finsotecc$snus_status, useNA = "ifany")
finsotecc$snus_status2 <- recode_factor(finsotecc$snus_status,
                                        "daily user" = "current user")
finsotecc$snus_status2 <- recode_factor(finsotecc$snus_status,
                                        "occasional" = "current user")

finsotecc$ecig_nic_status2 <- recode_factor(finsotecc$ecig_nic_status,
                                            "daily user" = "current user")
finsotecc$ecig_nic_status2 <- recode_factor(finsotecc$ecig_nic_status,
                                            "occasional" = "current user")
finsotecc$ecig_nonic_status2 <- recode_factor(finsotecc$ecig_nonic_status,
                                              "daily user" = "current user")
finsotecc$ecig_nonic_status2 <- recode_factor(finsotecc$ecig_nonic_status,
                                              "occasional" = "current user")
finsotecc$nrt_status2 <- recode_factor(finsotecc$nrt_status,
                                       "daily user" = "current user")
finsotecc$nrt_status2 <- recode_factor(finsotecc$nrt_status,
                                       "occasional" = "current user")

# Reference levels
finsotecc <- within(finsotecc, smoking_status <- relevel(smoking_status,
                                                         ref = 'never smoker'))
finsotecc <- within(finsotecc, snus_status <- relevel(snus_status,
                                                      ref = 'never user'))
finsotecc <- within(finsotecc, ecig_nic_status <- relevel(ecig_nic_status,
                                                          ref = 'never user'))
finsotecc <- within(finsotecc, ecig_nonic_status <- relevel(ecig_nonic_status,
                                                            ref = 'never user'))
finsotecc <- within(finsotecc, nrt_status <- relevel(nrt_status,
                                                     ref = 'never user'))

# Exports data into Stata
require(foreign)
write.dta(finsotecc, "finsote.dta")

#stata_src <- ''

# Setting up survey design (weights, strata and FPC)
```

```
des_final <- svydesign(id=~1, fpc=~rg_N_suomi, weights=~w_analysis_suomi,
                       strata=~rg_stratum_suomi, data=finsotecc)

des_final_males <- svydesign(id=~1, fpc=~rg_N_suomi, weights=~w_analysis_suomi,
                             strata=~rg_stratum_suomi, data=finsote_males)

des_final_females <- svydesign(id=~1, fpc=~rg_N_suomi, weights=~w_analysis_suomi,
                               strata=~rg_stratum_suomi, data=finsote_females)
```

Table 1

We used the function *svyCreateTableOne* from the package *tableone* to produce cross-tabulations that take the complex survey design into account

```
# Summary of sociodemographic characteristics of the whole sample (for text in Results)

listVars <- c("sex", "age_cont", "maritalstatus_bin", "educ_years",
              "mother_tongue", "involvement_attend_j")
catVars <- c("sex", "maritalstatus_bin", "mother_tongue")
table1 <- svyCreateTableOne(listVars, factor=catVars,
                            test=FALSE, data=des_final)
tab1 <- print(table1, printToggle=FALSE, nospaces=TRUE, format="p", contDigits=1)
kable(tab1, format.args=list(digits=1, nsmall=2, format))

# Table 1 - sociodemographic characteristics over time
table(finsotecc$dataid)
listVars <- c("sex", "age_cont", "maritalstatus_bin", "educ_years",
              "mother_tongue", "involvement_attend_j")
catVars <- c("sex", "maritalstatus_bin", "mother_tongue")
table1 <- svyCreateTableOne(listVars, strata=c("dataid"), factor=catVars,
                            test=FALSE, data=des_final)
tab1 <- print(table1, printToggle=FALSE, nospaces=TRUE, format="p", contDigits=1)
kable(tab1, format.args=list(digits=1, nsmall=2, format))

# Crude change (not marginal effects)
listVars <- c("smoking_status", "snus_status", "ecig_nic_status", "ecig_nonic_status",
              "nrt_status")
catVars <- c("smoking_status", "snus_status", "ecig_nic_status", "ecig_nonic_status",
             "nrt_status")
table1 <- svyCreateTableOne(listVars, strata=c("dataid"), factor=catVars, test=FALSE,
                            data=des_final)
tab1 <- print(table1, printToggle=FALSE, nospaces=TRUE, format="p", contDigits=1)
kable(tab1, format.args=list(digits=1, nsmall=1, format))

# Stratified by sex
## Men
listVars <- c("smoking_status", "snus_status", "ecig_nic_status", "ecig_nonic_status",
              "nrt_status")
catVars <- c("smoking_status", "snus_status", "ecig_nic_status", "ecig_nonic_status",
             "nrt_status")
table1 <- svyCreateTableOne(listVars, strata=c("dataid"), factor=catVars, test=FALSE,
                            data=des_final_males)
```

```r
tab1 <- print(table1, printToggle=FALSE, nospaces=TRUE, format="p", contDigits=1)
kable(tab1, format.args=list(digits=1, nsmall=1, format))

## Women
listVars <- c("smoking_status", "snus_status", "ecig_nic_status", "ecig_nonic_status",
              "nrt_status")
catVars <- c("smoking_status", "snus_status", "ecig_nic_status", "ecig_nonic_status",
             "nrt_status")
table1 <- svyCreateTableOne(listVars, strata=c("dataid"), factor=catVars, test=FALSE,
                            data=des_final_females)
tab1 <- print(table1, printToggle=FALSE, nospaces=TRUE, format="p", contDigits=1)
kable(tab1, format.args=list(digits=1, nsmall=1, format))

# Table S2 for snus
table(finsotecc$event, finsotecc$snus_status)
table(finsotecc$snus_status)

listVars <- c("sex", "age_cont", "maritalstatus_bin", "educ_years", "mother_tongue",
              "involvement_attend_j", "bmi")
catVars <- c("sex", "maritalstatus_bin", "mother_tongue")
table1 <- svyCreateTableOne(listVars, strata=c("snus_status"), factor=catVars,
                            test=FALSE, data=des_final)
tab1 <- print(table1, printToggle=FALSE, nospaces=TRUE, format="p", contDigits=1)
kable(tab1, format.args=list(digits=4, nsmall=2, format))

# Table S3 for e-cigarettes with nicotine
table(finsotecc$event, finsotecc$ecig_nic_status)
table(finsotecc$ecig_nic_status)

listVars <- c("sex", "age_cont", "maritalstatus_bin", "educ_years", "mother_tongue",
              "involvement_attend_j", "bmi")
catVars <- c("sex", "maritalstatus_bin", "mother_tongue")
table1 <- svyCreateTableOne(listVars, strata=c("ecig_nic_status"), factor=catVars,
                            test=FALSE, data=des_final)
tab1 <- print(table1, printToggle=FALSE, nospaces=TRUE, format="p", contDigits=1)
kable(tab1, format.args=list(digits=4, nsmall=2, format))

# Table S4 for e-cigarettes without nicotine
table(finsotecc$event, finsotecc$ecig_nonic_status)
table(finsotecc$ecig_nonic_status)

listVars <- c("sex", "age_cont", "maritalstatus_bin", "educ_years", "mother_tongue",
              "involvement_attend_j", "bmi")
catVars <- c("sex", "maritalstatus_bin", "mother_tongue")
table1 <- svyCreateTableOne(listVars, strata=c("ecig_nonic_status"), factor=catVars,
                            test=FALSE, data=des_final)
tab1 <- print(table1, printToggle=FALSE, nospaces=TRUE, format="p", contDigits=1)
kable(tab1, format.args=list(digits=4, nsmall=2, format))

# Table S5 for nicotine replacement therapy
table(finsotecc$event, finsotecc$nrt_status)
table(finsotecc$nrt_status)
```

```
listVars <- c("sex", "age_cont", "maritalstatus_bin", "educ_years", "mother_tongue",
              "involvement_attend_j", "bmi")
catVars <- c("sex", "maritalstatus_bin", "mother_tongue")
table1 <- svyCreateTableOne(listVars, strata=c("nrt_status"), factor=catVars, test=FALSE,
                            data=des_final)
tab1 <- print(table1, printToggle=FALSE, nospaces=TRUE, format="p", contDigits=1)
kable(tab1, format.args=list(digits=4, nsmall=2, format))
```

Main analyses

Main analyses were conducted in Stata SE v17. The reason for this was that none of the packages for marginal effects support ordered logistic regression models. We contacted Dr Vincent Arel-Bundock who was open to the idea of adapting the *marginaleffects* package for this purpose, but the *survey* package does not produce predicted estimates. Dr Arel-Bundock opened an issue in his GitHub and contacted Prof Thomas Lumley (mantainer of the *survey* package), but to date this issue has not been resolved.

We direct you to the file: Tobrisk changes.do for continuing the analyses.

We uploaded the output of the models into a Google Spreadsheet, which is read directly in the following section, to create the graphs using *ggplot2*.

Figure 1. Trends on daily smoking by sociodemographic groups

```
# Figure 1a. Smoking status (daily users)
figure1a <- read_sheet("https://docs.google.com/spreadsheets/d/1TvtzvGbf8PcNRNjcrMtjFEBds-qqEsLM7Vx_mI2

number_ticks <- function(n) {function(limits) pretty(limits, n)}

figure1a <- figure1a %>%
  filter(smoking_status==3) %>%
  mutate(prevalence=proportion*100) %>%
  mutate(lci2=lci*100) %>%
  mutate(uci2=uci*100) %>%
  mutate(category = fct_relevel(category, c("20-39 years old", "40-59 years old",
                                             "60 years and older", "Lowest tertile",
                                             "Middle tertile", "Highest tertile",
                                             "Married or cohabiting",
                                             "Separated, widowed or divorced",
                                             "Finnish", "Swedish", "Other",
                                             "No participation", "Occasional",
                                             "Active participation")))

ci <- aes(ymin=lci2, ymax=uci2)
dodge <- position_dodge(width=0.9)
dailysmoking <- ggplot(figure1a, aes(x=category, y=prevalence, fill=factor(year))) +
    geom_bar(stat="identity", position=dodge) +
    geom_errorbar(ci, position=dodge, width=0.20, size=0.20) +
    xlab("") +
    ylab("Prevalence") +
    labs(fill = "Year")  +
    scale_fill_jama() + theme_Publication() +
```

```
     theme(legend.position = "none", axis.text.x = element_blank(),
           axis.title.y = element_blank()) +
    scale_x_discrete(labels = function(category) str_wrap(category, width = 9)) +
    scale_y_continuous(breaks=c(0,4,8,12,16))
dailysmoking
ggsave(file="dailysmoking2.svg", units="cm", width=21, height=15, dpi=600)

#breaks = extended_breaks(n = 2)

# Figure 1b. Snus status (daily users)
figure1b <- read_sheet("https://docs.google.com/spreadsheets/d/1TvtzvGbf8PcNRNjcrMtjFEBds-qqEsLM7Vx_mI2

figure1b <- figure1b %>%
  filter(snus_status==2) %>%
  mutate(prevalence=proportion*100) %>%
  mutate(lci2=lci*100) %>%
  mutate(uci2=uci*100) %>%
 mutate(category = fct_relevel(category, c("20-39 years old", "40-59 years old",
                                           "60 years and older", "Lowest tertile",
                                           "Middle tertile", "Highest tertile",
                                           "Married or cohabiting", "Separated,
                                           widowed or divorced", "Finnish",
                                           "Swedish", "Other", "No participation",
                                           "Occasional", "Active participation")))

ci <- aes(ymin=lci2, ymax=uci2)
dodge <- position_dodge(width=0.9)
dailysnus <- ggplot(figure1b, aes(x=category, y=prevalence, fill=factor(year))) +
    geom_bar(stat="identity", position=dodge) +
    geom_errorbar(ci, position=dodge, width=0.20, size=0.20) +
    xlab("") +
    ylab("Prevalence") +
    labs(fill = "Year")  +
    scale_fill_jama() + theme_Publication() +
    theme(legend.position = "none", axis.text.x = element_blank(),
          axis.title.y = element_blank()) +
    scale_x_discrete(labels = function(category) str_wrap(category, width = 9)) +
    scale_y_continuous(breaks=c(0,1,2,3,4))
ggsave(file="dailysnus.svg", units="cm", width=21, height=15, dpi=600)

# Figure 1c. E-cig nicotine status (daily users)
figure1c <- read_sheet("https://docs.google.com/spreadsheets/d/1TvtzvGbf8PcNRNjcrMtjFEBds-qqEsLM7Vx_mI2

figure1c <- figure1c %>%
  filter(ecig_nic_status==2) %>%
  mutate(prevalence=proportion*100) %>%
  mutate(lci2=lci*100) %>%
  mutate(uci2=uci*100) %>%
  mutate(category = fct_relevel(category, c("20-39 years old", "40-59 years old",
                                            "60 years and older", "Lowest tertile",
                                            "Middle tertile", "Highest tertile",
                                            "Married or cohabiting", "Separated,
                                            widowed or divorced", "Finnish",
```

```
                                            "Swedish", "Other", "No participation",
                                            "Occasional", "Active participation")))

ci <- aes(ymin=lci2, ymax=uci2)
dodge <- position_dodge(width=0.9)
dailyecig_nic <- ggplot(figure1c, aes(x=category, y=prevalence, fill=factor(year))) +
    geom_bar(stat="identity", position=dodge) +
    geom_errorbar(ci, position=dodge, width=0.20, size=0.20) +
    xlab("") +
    ylab("Prevalence") +
    scale_fill_jama() + theme_Publication() +
     theme(legend.position = "none", axis.text.x = element_blank(),
           axis.title.y = element_blank()) +
    scale_x_discrete(labels = function(category) str_wrap(category, width = 9)) +
    scale_y_continuous(breaks=c(0,0.3,0.6,0.9,1.2))
ggsave(file="dailyecig_nic.svg", units="cm", width=21, height=15, dpi=600)

# Figure 1d. E-cig no nicotine status (daily users)
figure1d <- read_sheet("https://docs.google.com/spreadsheets/d/1TvtzvGbf8PcNRNjcrMtjFEBds-qqEsLM7Vx_mI2

figure1d <- figure1d %>%
  filter(ecig_nonic_status==2) %>%
  mutate(prevalence=proportion*100) %>%
  mutate(lci2=lci*100) %>%
  mutate(uci2=uci*100) %>%
  mutate(category = fct_relevel(category, c("20-39 years old", "40-59 years old",
                                            "60 years and older", "Lowest tertile", "Middle tertile", "
                                            "Swedish", "Other", "No participation",
                                            "Occasional", "Active participation")))

ci <- aes(ymin=lci2, ymax=uci2)
dodge <- position_dodge(width=0.9)
dailyecig_nonic <- ggplot(figure1d, aes(x=category, y=prevalence, fill=factor(year))) +
    geom_bar(stat="identity", position=dodge) +
    geom_errorbar(ci, position=dodge, width=0.20, size=0.20) +
    xlab("") +
    ylab("Prevalence") +
    scale_fill_jama() + theme_Publication() +
     theme(legend.position = "none", axis.text.x = element_blank(),
           axis.title.y = element_blank()) +
    scale_x_discrete(labels = function(category) str_wrap(category, width = 9)) +
    scale_y_continuous(breaks=c(0,0.04, 0.08, 0.12, 0.16))
ggsave(file="dailyecig_nonic.svg", units="cm", width=21, height=15, dpi=600)


# Figure 1e. Any daily tobacco or nicotine use
figure1e <- read_sheet("https://docs.google.com/spreadsheets/d/1TvtzvGbf8PcNRNjcrMtjFEBds-qqEsLM7Vx_mI2

figure1e <- figure1e %>%
  filter(any_tobaccodaily==2) %>%
  mutate(prevalence=proportion*100) %>%
  mutate(lci2=lci*100) %>%
  mutate(uci2=uci*100) %>%
```

```
  mutate(category = fct_relevel(category, c("20-39 years old", "40-59 years old",
                                           "60 years and older", "Lowest tertile",
                                           "Middle tertile", "Highest tertile",
                                           "Married or cohabiting", "Separated,
                                           widowed or divorced", "Finnish",
                                           "Swedish", "Other", "No participation",
                                           "Occasional", "Active participation")))

ci <- aes(ymin=lci2, ymax=uci2)
dodge <- position_dodge(width=0.9)
dailyany <- ggplot(figure1e, aes(x=category, y=prevalence, fill=factor(year))) +
    geom_bar(stat="identity", position=dodge) +
    geom_errorbar(ci, position=dodge, width=0.20, size=0.20) +
    xlab("") +
    ylab("Prevalence") +
    scale_fill_jama() + theme_Publication() +
    theme(legend.position = "none", axis.text.x = element_blank(),
          axis.title.y = element_blank()) +
    scale_x_discrete(labels = function(category) str_wrap(category, width = 9)) +
    scale_y_continuous(breaks=c(0,6,12,18,24))
ggsave(file="dailyany.svg", units="cm", width=21, height=10, dpi=600)

# Figure 1f. NRT status (daily users)
figure1f <- read_sheet("https://docs.google.com/spreadsheets/d/1TvtzvGbf8PcNRNjcrMtjFEBds-qqEsLM7Vx_mI2

figure1f <- figure1f %>%
  filter(nrt_status==2) %>%
  mutate(prevalence=proportion*100) %>%
  mutate(lci2=lci*100) %>%
  mutate(uci2=uci*100) %>%
  mutate(category = fct_relevel(category, c("20-39 years old", "40-59 years old",
                                           "60 years and older", "Lowest tertile",
                                           "Middle tertile", "Highest tertile",
                                           "Married or cohabiting", "Separated,
                                           widowed or divorced", "Finnish",
                                           "Swedish", "Other", "No participation",
                                           "Occasional", "Active participation")))

ci <- aes(ymin=lci2, ymax=uci2)
dodge <- position_dodge(width=0.9)
dailynrt <- ggplot(figure1f, aes(x=category, y=prevalence, fill=factor(year))) +
    geom_bar(stat="identity", position=dodge) +
    geom_errorbar(ci, position=dodge, width=0.20, size=0.20) +
    xlab("") +
    ylab("Prevalence") +
    scale_fill_jama() + theme_Publication() +
     theme(legend.position = "none", axis.text.x = element_blank(),
           axis.title.y = element_blank()) +
    scale_x_discrete(labels = function(category) str_wrap(category, width = 9)) +
    scale_y_continuous(breaks=c(0,0.5, 1, 1.5, 2))
ggsave(file="dailynrt.svg", units="cm", width=21, height=10, dpi=600)

# Merging all figures into a single one
```

```
g1 <- ggplotGrob(dailysmoking)
g2 <- ggplotGrob(dailysnus)
g3 <- ggplotGrob(dailyecig_nic)
g4 <- ggplotGrob(dailyecig_nonic)
g5 <- ggplotGrob(dailyany)
g6 <- ggplotGrob(dailynrt)
maxWidth = grid::unit.pmax(g1$widths[2:4], g2$widths[2:4], g3$widths[2:4],
                           g4$widths[2:4],
                           g5$widths[2:4], g6$widths[2:4])
g1$widths[2:4] <- as.list(maxWidth)
g2$widths[2:4] <- as.list(maxWidth)
g3$widths[2:4] <- as.list(maxWidth)
g4$widths[2:4] <- as.list(maxWidth)
g5$widths[2:4] <- as.list(maxWidth)
g6$widths[2:4] <- as.list(maxWidth)
dailyplot <- grid.arrange(g1, g2, g3, g4, g5, g6, nrow=6)
ggsave("testplot.svg", units="cm", width=21, height=27, dpi=600, dailyplot)
```

Figure S1. Trends on occasional smoking by sociodemographic groups

```
# Figure 1a. Smoking status (occasional users)
figure1a <- read_sheet("https://docs.google.com/spreadsheets/d/1TvtzvGbf8PcNRNjcrMtjFEBds-qqEsLM7Vx_mI2

number_ticks <- function(n) {function(limits) pretty(limits, n)}

figure1a <- figure1a %>%
  filter(smoking_status==2) %>%
  mutate(prevalence=proportion*100) %>%
  mutate(lci2=lci*100) %>%
  mutate(uci2=uci*100) %>%
  mutate(category = fct_relevel(category, c("20-39 years old", "40-59 years old",
                                            "60 years and older", "Lowest tertile",
                                            "Middle tertile", "Highest tertile",
                                            "Married or cohabiting", "Separated,
                                            widowed or divorced", "Finnish",
                                            "Swedish", "Other", "No participation",
                                            "Occasional", "Active participation")))

ci <- aes(ymin=lci2, ymax=uci2)
dodge <- position_dodge(width=0.9)
occasionalsmoking <- ggplot(figure1a, aes(x=category, y=prevalence,
                                          fill=factor(year))) +
    geom_bar(stat="identity", position=dodge) +
    geom_errorbar(ci, position=dodge, width=0.20, size=0.20) +
    xlab("") +
    ylab("Prevalence") +
    labs(fill = "Year")  +
    scale_fill_jama() + theme_Publication() +
     theme(legend.position = "none", axis.text.x = element_blank(),
           axis.title.y = element_blank()) +
    scale_x_discrete(labels = function(category) str_wrap(category, width = 9)) +
```

```
    scale_y_continuous(breaks=c(0,2.5,5,7.5,10))
occasionalsmoking
ggsave(file="occasionalsmoking.svg", units="cm", width=21, height=15, dpi=600)

#breaks = extended_breaks(n = 2)

# Figure 1b. Snus status (occasional users)
figure1b <- read_sheet("https://docs.google.com/spreadsheets/d/1TvtzvGbf8PcNRNjcrMtjFEBds-qqEsLM7Vx_mI2

figure1b <- figure1b %>%
  filter(snus_status==3) %>%
  mutate(prevalence=proportion*100) %>%
  mutate(lci2=lci*100) %>%
  mutate(uci2=uci*100) %>%
 mutate(category = fct_relevel(category, c("20-39 years old", "40-59 years old",
                                           "60 years and older", "Lowest tertile",
                                           "Middle tertile", "Highest tertile",
                                           "Married or cohabiting", "Separated,
                                           widowed or divorced", "Finnish", "Swedish",
                                           "Other", "No participation", "Occasional",
                                           "Active participation")))

ci <- aes(ymin=lci2, ymax=uci2)
dodge <- position_dodge(width=0.9)
occasionalsnus <- ggplot(figure1b, aes(x=category, y=prevalence, fill=factor(year))) +
    geom_bar(stat="identity", position=dodge) +
    geom_errorbar(ci, position=dodge, width=0.20, size=0.20) +
    xlab("") +
    ylab("Prevalence") +
    labs(fill = "Year")  +
    scale_fill_jama() + theme_Publication() +
    theme(legend.position = "none", axis.text.x = element_blank(),
          axis.title.y = element_blank()) +
    scale_x_discrete(labels = function(category) str_wrap(category, width = 9)) +
    scale_y_continuous(breaks=c(0,1,2,3,4))
occasionalsnus
ggsave(file="occasionalnus.svg", units="cm", width=21, height=15, dpi=600)

# Figure 1c. E-cig nicotine status (occasional users)
figure1c <- read_sheet("https://docs.google.com/spreadsheets/d/1TvtzvGbf8PcNRNjcrMtjFEBds-qqEsLM7Vx_mI2

figure1c <- figure1c %>%
  filter(ecig_nic_status==3) %>%
  mutate(prevalence=proportion*100) %>%
  mutate(lci2=lci*100) %>%
  mutate(uci2=uci*100) %>%
  mutate(category = fct_relevel(category, c("20-39 years old", "40-59 years old",
                                            "60 years and older", "Lowest tertile",
                                            "Middle tertile", "Highest tertile",
                                            "Married or cohabiting", "Separated,
                                            widowed or divorced", "Finnish",
                                            "Swedish", "Other", "No participation",
                                            "Occasional", "Active participation")))
```

```
ci <- aes(ymin=lci2, ymax=uci2)
dodge <- position_dodge(width=0.9)
occasionalecig_nic <- ggplot(figure1c, aes(x=category, y=prevalence,
                                           fill=factor(year))) +
    geom_bar(stat="identity", position=dodge) +
    geom_errorbar(ci, position=dodge, width=0.20, size=0.20) +
    xlab("") +
    ylab("Prevalence") +
    scale_fill_jama() + theme_Publication() +
     theme(legend.position = "none", axis.text.x = element_blank(),
           axis.title.y = element_blank()) +
    scale_x_discrete(labels = function(category) str_wrap(category, width = 9)) +
    scale_y_continuous(breaks=c(0,0.4,0.8,1.2,1.6))
occasionalecig_nic
ggsave(file="occasionalecig_nonic.svg", units="cm", width=21, height=15, dpi=600)

# Figure 1d. E-cig no nicotine status (occasional users)
figure1d <- read_sheet("https://docs.google.com/spreadsheets/d/1TvtzvGbf8PcNRNjcrMtjFEBds-qqEsLM7Vx_mI2

figure1d <- figure1d %>%
  filter(ecig_nonic_status==3) %>%
  mutate(prevalence=proportion*100) %>%
  mutate(lci2=lci*100) %>%
  mutate(uci2=uci*100) %>%
  mutate(category = fct_relevel(category, c("20-39 years old", "40-59 years old",
                                            "60 years and older", "Lowest tertile",
                                            "Middle tertile", "Highest tertile",
                                            "Married or cohabiting", "Separated,
                                            widowed or divorced", "Finnish",
                                            "Swedish", "Other", "No participation",
                                            "Occasional", "Active participation")))

ci <- aes(ymin=lci2, ymax=uci2)
dodge <- position_dodge(width=0.9)
occasionalecig_nonic <- ggplot(figure1d, aes(x=category, y=prevalence,
                                             fill=factor(year))) +
    geom_bar(stat="identity", position=dodge) +
    geom_errorbar(ci, position=dodge, width=0.20, size=0.20) +
    xlab("") +
    ylab("Prevalence") +
    scale_fill_jama() + theme_Publication() +
     theme(legend.position = "none", axis.text.x = element_blank(),
           axis.title.y = element_blank()) +
    scale_x_discrete(labels = function(category) str_wrap(category, width = 9)) +
    scale_y_continuous(breaks=c(0,0.2, 0.4, 0.6, 0.8))
occasionalecig_nonic
ggsave(file="occasionalecig_nonic.svg", units="cm", width=21, height=15, dpi=600)

# Figure 1e. NRT status (occasional users)
figure1e <- read_sheet("https://docs.google.com/spreadsheets/d/1TvtzvGbf8PcNRNjcrMtjFEBds-qqEsLM7Vx_mI2

figure1e <- figure1e %>%
  filter(nrt_status==2) %>%
```

```r
  mutate(prevalence=proportion*100) %>%
  mutate(lci2=lci*100) %>%
  mutate(uci2=uci*100) %>%
  mutate(category = fct_relevel(category, c("20-39 years old", "40-59 years old",
                                            "60 years and older", "Lowest tertile",
                                            "Middle tertile", "Highest tertile",
                                            "Married or cohabiting", "Separated,
                                            widowed or divorced", "Finnish",
                                            "Swedish", "Other", "No participation",
                                            "Occasional", "Active participation")))

ci <- aes(ymin=lci2, ymax=uci2)
dodge <- position_dodge(width=0.9)
occasionalnrt <- ggplot(figure1e, aes(x=category, y=prevalence,
                                      fill=factor(year))) +
    geom_bar(stat="identity", position=dodge) +
    geom_errorbar(ci, position=dodge, width=0.20, size=0.20) +
    xlab("") +
    ylab("Prevalence") +
    scale_fill_jama() + theme_Publication() +
     theme(legend.position = "none", axis.text.x = element_blank(),
           axis.title.y = element_blank()) +
    scale_x_discrete(labels = function(category) str_wrap(category, width = 9)) +
    scale_y_continuous(breaks=c(0,0.5, 1, 1.5, 2))
occasionalnrt
ggsave(file="occasionalnrt.svg", units="cm", width=21, height=15, dpi=600)

# Merging all figures into a single one. The code aligns the Y axes even if the Y-scales have different
g1 <- ggplotGrob(occasionalsmoking)
g2 <- ggplotGrob(occasionalsnus)
g3 <- ggplotGrob(occasionalecig_nic)
g4 <- ggplotGrob(occasionalecig_nonic)
g5 <- ggplotGrob(occasionalnrt)
maxWidth = grid::unit.pmax(g1$widths[2:4], g2$widths[2:4], g3$widths[2:4],
                           g4$widths[2:4],
                           g5$widths[2:4])
g1$widths[2:4] <- as.list(maxWidth)
g2$widths[2:4] <- as.list(maxWidth)
g3$widths[2:4] <- as.list(maxWidth)
g4$widths[2:4] <- as.list(maxWidth)
g5$widths[2:4] <- as.list(maxWidth)
occasionalplot <- grid.arrange(g1, g2, g3, g4, g5, nrow=6)
ggsave("occasionalplot.svg", units="cm", width=21, height=27, dpi=600, occasionalplot)
```

End of script

Please contact me if you find errors or have any feedback on the code at @spenafajuri (Twitter) or sebastian.penafajuri@thl.fi
